# Targeted delivery of a STING agonist to brain tumors using bioengineered protein nanoparticles for enhanced immunotherapy

**DOI:** 10.1016/j.bioactmat.2022.02.026

**Published:** 2022-03-01

**Authors:** Bin Wang, Maoping Tang, Ziwei Yuan, Zhongyu Li, Bin Hu, Xin Bai, Jinxian Chu, Xiaoyang Xu, Xue-Qing Zhang

**Affiliations:** a Engineering Research Center of Cell & Therapeutic Antibody, Ministry of Education, School of Pharmacy, Shanghai Jiao Tong University, 800 Dongchuan Road, Shanghai, 200240, PR China; b Department of Chemical and Materials Engineering, New Jersey Institute of Technology, Newark, NJ, 07102, USA

**Keywords:** Blood brain barrier, Bioengineered protein nanoparticles, Dual-targeting property, STING agonist, Glioma-targeted immunotherapy

## Abstract

Immunotherapy is emerging as a powerful tool for combating many human diseases. However, the application of this life-saving treatment in serious brain diseases, including glioma, is greatly restricted. The major obstacle is the lack of effective technologies for transporting therapeutic agents across the blood-brain barrier (BBB) and achieving targeted delivery to specific cells once across the BBB. Ferritin, an iron storage protein, traverses the BBB via receptor-mediated transcytosis by binding to transferrin receptor 1 (TfR1) overexpressed on BBB endothelial cells. Here, we developed bioengineered ferritin nanoparticles as drug delivery carriers that enable the targeted delivery of a small-molecule immunomodulator to achieve enhanced immunotherapeutic efficacy in an orthotopic glioma-bearing mouse model. We fused different glioma-targeting moieties on self-assembled ferritin nanoparticles via genetic engineering, and RGE fusion protein nanoparticles (RGE-HFn NPs) were identified as the best candidate. Furthermore, RGE-HFn NPs encapsulating a stimulator of interferon genes (STING) agonist (SR717@RGE-HFn NPs) maintained stable self-assembled structure and targeting properties even after traversing the BBB. In the glioma-bearing mouse model, SR717@RGE-HFn NPs elicited a potent local innate immune response in the tumor microenvironment, resulting in significant tumor growth inhibition and prolonged survival. Overall, this biomimetic brain delivery platform offers new opportunities to overcome the BBB and provides a promising approach for brain drug delivery and immunotherapy in patients with glioma.

## Highlights

RGE-HFn NPs showed excellent glioma-targeting ability.
RGE-HFn NPs showed potent tumor tissue-penetration ability.
SR717@RGE-HFn NPs effectively activated the STING pathway and exerted immunoregulatory effects within the intracranial glioma TME.
SR717@RHE-HFn NPs significantly triggered a glioma-specific innate immune response and remarkably delayed the growth of orthotopic gliomas without exhibiting apparent systemic toxicity.

## 1 Introduction

Glioma is one of the most common and aggressive primary brain tumors. Even with the standard medical approach of surgical resection of the primary tumor, adjuvant radiotherapy and systemic chemotherapy with temozolomide (TMZ) [1,2], the median survival time of patients with glioma is only 14.6 months, and the five-year survival rate is still less than 10% [2]. The enormous challenges in glioma treatment include the highly heterogeneous and dense structure within tumor tissue that hinders the transport of agents across the tumor tissue compartment [3,4], as well as the intrinsic resistance of tumor cells mediated by blood-brain barrier (BBB) protection [[5], [6], [7]]. The BBB, which is a biological system composed of astrocytes, neurons, pericytes and vascular endothelial cells, represents a protective interface between the CNS and the blood that prevents the entry of exogenous toxins and pathogens into the brain [8]. However, the highly selective permeability of the BBB also inhibits the passage of the vast majority of small-molecule drugs and macromolecules (e.g., peptides, genes, and protein therapeutics), which is a major obstacle facing brain disease treatment [[9], [10], [11]].Therefore, there exists a tremendous demand for delivery systems that transport molecules across the BBB, penetrate deep into the tumor and specifically target cells of interest once past the BBB.

Glioma grows in an immunocompromised environment characterized by high densities of immunosuppressive cells with inhibitory potential for anti-glioma immunity and a lack of sufficient T cell infiltration [12,13]. These features have prompted the demand for strategies to reprogram glioma-associated immunosuppression to immunogenic and pro-inflammatory states that revive glioma-directed immune responses. Activation of stimulator of interferon genes (STING), an endoplasmic reticulum-associated homodimeric protein, triggers a signaling cascade through tank-binding kinase 1 (TBK1)/interferon regulatory factor 3 (IRF3) and nuclear factor kappa-B (NF-κB), which culminates in increased synthesis and secretion of type I interferons (IFNs) and proinflammatory cytokines [[14], [15], [16]]. Type I IFNs stimulate T cell cross-priming and increase the infiltration of natural killer (NK) cells and dendritic cells (DCs) into tumors, which are essential for the development of robust antitumor adaptive immunity [[17], [18], [19]]. Although small molecules that activate the STING pathway could be a potentially promising strategy to improve immunotherapeutic efficiency in glioma, systemically administered STING agonists might induce undesired inflammation throughout the body due to the ubiquitous expression of STING in both tumor and normal tissues, which limits clinical administration to intratumoral routes only. The development of drug delivery systems capable of traversing the BBB and improving the homing of cargos to target cells is crucial to address this problem.

Considerable efforts have been made to develop various drug carriers, including liposomes, cationic polymers and inorganic nanoparticles for improving drug delivery across the BBB, but limited success has been achieved in the translation from bench to bedside due to complicated production procedures, low delivery efficacy, lack of specificity, and uncertain safety issues associated with nanomaterials and surfactants [[20], [21], [22]]. Natural substances (e.g., human ferritin nanoparticles, etc.) that cross the BBB emerged throughout evolutionary processes and therefore have an innate biocompatibility and efficacy. Leveraging their natural properties and biological interactions with the BBB might provide critical insights into the future development and clinical translation of bioinspired brain-targeted delivery systems. Human heavy-chain ferritin nanoparticles (HFn NPs) composed of 24 heavy-chain ferritin subunits are characterized by a globular structure whose interior cavity diameter is 8 nm and the outer diameter is 12 nm [23]. As a natural iron storage protein, ferritin shows excellent biocompatibility and biodegradability [24]. According to recent studies, HFn NPs traverse the BBB through receptor-mediated transcytosis by binding to transferrin receptor 1 (TfR1), which is overexpressed on BBB endothelial cells [25]. Yan et al. explored the applications of HFn NPs for drug delivery to both subcutaneous and brain tumors [25,26]. HFn NPs have shown promise for brain drug delivery; however, few studies have focused on investigating their capabilities in promoting penetration into the tumor tissue and targeted delivery to glioma cells after traversing the BBB.

Here, we first fused glioma-targeting motifs on the surface of HFn NPs through a genetic engineering method and constructed a STING agonist-loaded fusion protein NP platform capable of penetrating deep tumor tissues and targeting gliomas after traversing the BBB. Several peptide ligands have been developed to actively target glioma cells. The RGERPPR motif shows a high binding affinity for NRP-1, a transmembrane glycoprotein overexpressed on glioma cells [27,28]. Pep-1 (CGEMGWVRC) is a specific ligand of interleukin 13 receptor a2 (IL-13Ra2), which is one of the subunits of the IL-13 receptor expressed on glioma cells [29,30]. The CGKRK peptide homes to epidermal tumors in mice, as evidenced by the strong accumulation of rhodamine-labeled CGKRK peptide in glioma tumors [31,32]. Among the different biosynthesized fusion proteins, an RGE peptide-fused protein successfully self-assembled into NPs (RGE-HFn NPs) and exhibited an excellent glioma-homing property and tissue penetrating ability while maintaining the existing ability of ferritin to traverse the BBB. Since cyclic dinucleotide-based STING agonists display limited cellular uptake, intrinsic instability and a short blood circulation half-life [33,34], we further incorporated a non-nucleotide STING agonist into the cavity of RGE-HFn NPs to enhance its delivery into intracranial glioma sites and improve therapeutic outcomes. Notably, the globular structure of HFn NPs disassembles into individual subunits when the pH is lowered to 2–3 but reassembles in a nearly intact manner when the pH returns to physiological conditions [35,36]. As the pH of normal tissue and blood circulation are neutral and the intracellular endosomal environment is acidic, this pH-mediated reversible assembly-disassembly property further expands the capacity of the RGE-HFn nanosystem for drug loading and delivery.

**Scheme 1:**
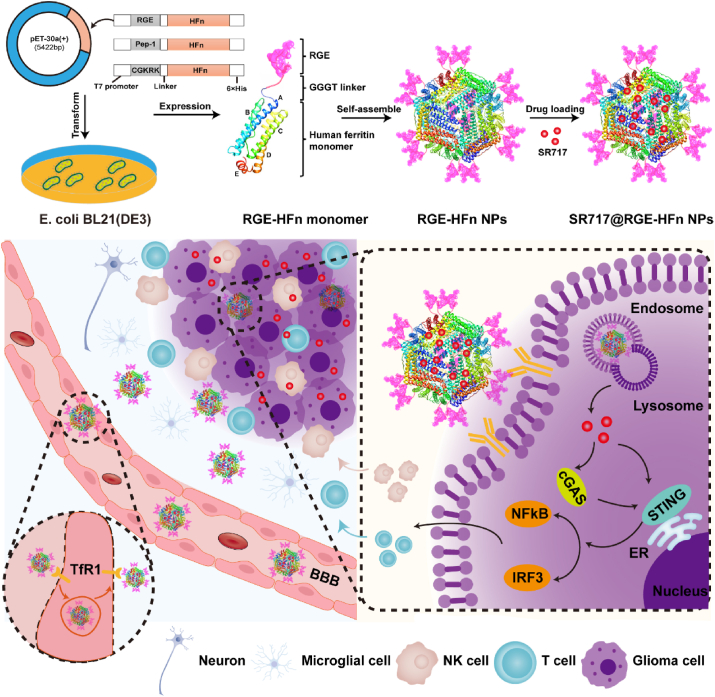
Schematic depiction of SR717@RGE-HFn NPs with a dual-targeting strategy for potent anti-glioma immune response. 3D model of the peptide-fused ferritin subunit and self-assembled peptide-HFn NPs were generated using Modellar (v 9.2) simulation software. For example, the RGE peptide fused to the ferritin subunit (blue) through a GGGT linker peptide was modeled based on the RGE peptide sequence (RGERPPR) and wtFH cage structure (PDB 2FHA). The peptide-engineered HFn NPs traverse the BBB through TfR1-mediated transcytosis. Once across the BBB, the tumor penetration peptide RGE facilitates deep penetration of NPs into the tumor tissue and effective homing of STING agonists to the glioma, leading to activation of the STING pathway.

For the first time, we successfully delivered a non-nucleotide STING agonist (SR717) [37] into an orthotopic mouse model of brain tumors via intravenous administration of engineered RGE-HFn NPs (Scheme 1). The durable accumulation of SR717 within the glioma tumor microenvironment (TME) resulted in the induction of a local innate immune response, as indicated by the significantly upregulated expression of STING signaling-related proteins, elevated mRNA levels of proinflammatory cytokines, and improved recruitment of CD8^+^ T cells, NK cells and DCs into the tumor tissue. As a result, potent inhibition of glioma growth and improved survival of glioma-bearing mice were achieved without causing any observable adverse effects on blood biochemical indicators or pathology of major organs. In conclusion, we developed a novel and simple drug delivery system with a dual-targeting mechanism that allows deep penetration into the tumor tissue and effective homing of STING agonists to the glioma once across the BBB, providing a promising immunotherapeutic strategy for the treatment of glioma.

## 2 Materials and methods

### 2.1 Materials

The hFTH gene (HG13217-G) was obtained as a cDNA clone from Sino Biological Inc. (China). pET-30a(+) plasmid was obtained from Hunan Fenghui Biotechnology Co., Ltd. (China). Isopropyl-*β*-D-thiogalactoside (IPTG), FITC and Cy5.5 N-hydroxysuccinimide (NHS) ester were purchased from Sigma-Aldrich Co., Ltd. (USA). Kanamycin, ammonium sulfate and gelatin were purchased from Shanghai Aladdin Bio-Chem Technology Co., Ltd. (China). SR717 was purchased from Shanghai Bidepharmatech Co., Ltd. (China). Dulbecco's modified Eagle's medium (DMEM), fetal bovine serum (FBS), Trypsin-EDTA, and Penicillin-Streptomycin solution were purchased from Gibco (USA). Unmentioned agents were purchased from Sigma-Aldrich (USA) unless otherwise indicated.

Akata start (GE, USA) was used to purify the target proteins. HPLC (Agilent, USA) was used to determine the loading and release of SR717. A transmission electron microscope (TEM, Thermo Scientific Talos, USA) was used to characterize the morphology of NPs. Dynamic light scattering (DLS, Malvern, UK) was used to characterize the mean particle size and zeta potential of NPs.

### 2.2 Cell lines and animals

RAW, THP-1, Luc-GL261 and bEnd.3 cells were obtained from the American Type Culture Collection (ATCC). G422 cell was purchased from the cell bank of the Committee on Type Culture Collection of the Chinese Academy of Sciences (CTCC, Shanghai, China). RAW, G422 and Luc-GL261 and bEnd.3 cells were cultured in DMEM medium containing 10% fetal calf serum, while THP-1 cell was cultured in RPMI 1640 medium containing 10% fetal calf serum. Male ICR or C57BL/6 mice were obtained from the Charles River Laboratories (Beijing). All animal studies were performed under the approval of the Animal Care and Use Committee of Shanghai Jiao Tong University.

### 2.3 Construction of RGE/Pep-1/CGKRK-HFn expression plasmids

HFn and RGE/Pep-1/CGKRK peptide-linker were prepared by PCR amplification of gene clones encoding NH_2_-NdeI-hFTH-6 × His-BamHI-COOH, NH_2_-NdeI-RGE/Pep-1/CGKRK-linker peptide (GGGGT)-COOH and NH_2_-linker peptide (GGGGT)-hFTH-6 × His-BamHI-COOH using appropriate primers. The NH_2_-NdeI-RGE/Pep-1/CGKRK-linker-HFn-6 × His –COOH was cloned using an overlap and extension PCR method. Each gene clone was ligated into a pET-30a(+) plasmid to yield the expression vectors, pET-HFn, pET-RGE/Pep-1/CGKRK-linker-HFn. The constructed vectors were subsequently transformed into *E. coli* BL21(DE3), and transformants were obtained by kanamycin-resistance selection.

### **2.4** Biosynthesis and characterization of HFn and peptide-HFn NPs

HFn and RGE/Pep-1/CGKRK-HFn were produced according to previously described procedures [38], but with significant modification and optimization in the preparation and purification processes of target NPs. Briefly, the expression vectors pET-HFn and pET-RGE/Pep-1/CGKRK-linker-HFn were transformed into *E. coli* BL21(DE3) according to the manufacturer's instructions. HFn and RGE/Pep-1/CGKRK-HFn proteins were expressed in *E. coli* BL21(DE3) where they self-assembled into the 24 subunit nanocages. The transformed *E. coli* cells were grown overnight in LB medium with 50 mg/L kanamycin. Then, the production of HFn and RGE/Pep-1/CGKRK-HFn proteins was induced by IPTG, and cells were incubated for an additional 4 h. After incubation, *E. coli* cells were harvested by centrifugation at 5000*g* for 45 min and the pellets were re-suspended in PBS buffer (50 mM NaH2PO4, 150 mM NaCl, pH 7.4). The re-suspended *E. coli* cells were sonicated on ice and centrifuged at 10,000 g for 30 min. The supernatant was heated at 65 °C for 15 min to denature and precipitate most *E. coli* proteins. After centrifugation, the HFn and RGE/Pep-1/CGKRK-HFn proteins in the supernatant were precipitated by ammonium sulfate (520 g/L). The precipitate was collected by centrifugation at 22,000 g for 45 min, and then dissolved in PBS buffer. After dialyzing out the ammonium sulfate, HFn and RGE/Pep-1/CGKRK-HFn proteins protein was purified by size exclusion chromatography on a Superdex 200 PG XK 16/100 column (GE Healthcare, USA) followed by Ni + affinity chromatography on HiTrap affinity column (GE Healthcare, USA). The concentration of HFn and RGE/Pep-1/CGKRK-HFn proteins were determined in triplicate by the bicinchoninic acid (BCA) protein assay kit (Beyotime Biotechnology, China) using bovine serum albumin as the standard. The typical yield of HFn and peptide-HFn fusion proteins were 10–100 mg per 1 L patch.

The yielded HFn, peptide-HFn and SR-717@RGE-HFn NPs were characterized using TEM for morphology analysis and DLS for mean particle size and zeta potential characterization.

### 2.5 Matrix-assisted laser desorption/ionization time-of-flight mass spectrometry (MALDI-TOF-MS)

The separated HFn, RGE-HFn, Pep-1-HFn or CGKRK-HFn subunits were obtained via 10% SDS-PAGE. 0.5 μL of the resulting samples were deposited on the plate and dried. CHCA (10 mg/mL) dissolved in 50% ACN/0.1% TFA was used as matrix. The molecular weights of subunits were measured in the positive ion and reflector modes using MALDI-TOF 7090 (Shimadzu, Kyoto).

### 2.6 Drug encapsulation in HFn NPs

SR717 or Doxorubicin (Dox) was encapsulated within HFn NPs or RGE-HFn NPs using a modified disassembly/reassembly method [39,40]. Briefly, suspensions of HFn NPs or RGE-HFn NPs were dissolved in 0.1 M NaCl and the pH was adjusted to 2.0 by adding HCl. SR717 or Dox was then added to the solution at a 300:1 mol ratio, and the pH was maintained at 2.5 for 20 min. The pH was then increased to 8.0 using 1 M NaOH. The resulting solution was stirred at room temperature for 2h and dialyzed against PBS at pH 7.4 to remove the unencapsulated SR717 or Dox. After dialysis, solutions were concentrated through 30 kDa Amicon ultrafiltration devices followed by sterile filtration, and stored at 4 °C prior to use.

### **2.7** *Cell binding studies*

The reactivity of RGE/Pep-1/CGKRK-HFn or HFn with G422 or GL261 cells was assessed by flow cytometry. Briefly, 20 μM FITC-conjugated peptide-HFn NPs were incubated with 100 μl detached cell suspensions (1 × 10^6^ cells per ml) for 4 h in PBS containing 0.3% bovine serum albumin. After three washes in PBS, cells were analyzed immediately using a FACS Calibur flow cytometry system (BD Biosciences, USA) and laser scanning confocal microscopy (LSCM, Leica, USA).

### **2.8** *Labeling of HFn and peptide-HFn NPs*

Cy5.5-labeled HFn and peptide-HFn were prepared by conjugating Cy5.5 NHS ester onto the surface lysine of the ferritin protein shell. Briefly, 100 nM Cy5.5 NHS ester in 5 μL dimethyl sulfoxide (DMSO) was added to a 50 nM HFn or peptide-HFn solution in 1 mL PBS buffer. The mixture was incubated at 4 °C overnight followed by dialysis against PBS to get rid of the unreacted agents. The concentration of Cy5.5 was determined by fluorescence spectroscopy (FL 6500, PerkinElmer, USA). The protein concentration was measured by the BCA protein assay kit using bovine serum albumin as the standard. The final mole ratio of Cy5.5: HFn was about 20:1.

Similarly, 200 nM FITC was added to a 50 nM HFn or peptide-HFn solution in 1 mL carbonate/bicarbonate buffer (100 mM carbonate, pH 9.0). The mixture was incubated at room temperature for 2 h followed by dialysis against PBS to get rid of the unreacted agents. The final mole ratio of FITC:HFn was determined as described.

### 2.9 Fluorescence-based ELISA

For fluorescence-based ELISA analysis, each well of a 96-well plate was pre-coated with 100 μL (0.25 mg/mL) recombinant murine TfR1 in PBS overnight at 4 °C. On the second day, the plate was blocked with 3% BSA in PBST, and then FITC-labeled HFn or peptide-HFn NPs (at the final concentration range of 0.1–10000 nM) was added into each well and incubated at room temperature for 1.5 h. After being washed twice with PBST, the relative FITC fluorescence intensity was determined by fluorescence spectroscopy (488 nm excitation and 515 nm emission).

### 2.10 In vitro BBB model and transcytosis assay

The *in vitro* BBB model was established with bEnd.3 cells using a transwell cell culture system. Briefly, bEnd.3 cells (1 × 10^5^ cells/well) were seeded onto the upper chamber of the transwell pre-coated with gelatin (2%), and cultured with DMEM medium containing 10% FBS. The integrity of the cell monolayer was evaluated by measuring the TEER values using a Millicell-ERS Volt-Ohm Meter (Millipore, Burlington, MA). The cell monolayers with TEER values higher than 200 Ω cm^2^ were used as the BBB model for the transcytosis assay. FITC-labeled HFn or peptide-HFn NPs were then added to the upper chamber. After 2 h incubation, samples from the basal chamber were collected to determine the NP concentration based on the standard curve generated by plotting protein concentrations against FITC fluorescence intensities (ex/em 490/525 nm). The integrity of the samples collected from the basal chamber was also analyzed by native polyacrylamide gel electrophoresis (PAGE, 5% polyacrylamide gels) using freshly prepared FITC-HFn NPs as control.

### 2.11 Evaluation of tumor spheroid penetration

G422 or GL261 cells were used to generate 3D glioma spheroids by a modified hanging drop method [41,42]. Briefly, we prepared an 80% culture medium containing 0.24% methylcellulose for spheroid growth. Each drop (20μL/drop) of the 0.24% methylcellulose-culture medium solution that contained 20,000 G422 or GL261 cells was pipetted onto the lid of a 100 mm dish and was hung over dishes containing PBS. Hanging drop cultures were incubated for 7 days to form cell aggregates. The resultant spheroids were gently harvested under sterile conditions and subsequently exposed to 1 μg/mL FITC-HFn or FITC-RGE-HFn for 6 h. 3D glioma spheroids were fixed with 4% paraformaldehyde and visualized by LSCM (Leica, Buffalo Grove, IL).

### 2.12 Cell viability assay

THP-1 or RAW cells were seeded at a density of 1 × 10^4^ cells/well in a 96-well plate overnight and incubated with escalating doses of free SR717 or SR717@RGE-HFn NPs for 24 h. Cells without treatment were used as control. The medium was then replaced with CCK8 reagent-containing complete medium, followed by incubation at 37 °C for 2 h. Absorbance at 490 nm was then measured using a multimodal plate reader (Tecan, Switzerland). The values were blank-subtracted and normalized to the untreated cell values to give relative cell viability.

### 2.13 Western blotting

Quick-frozen brain tissues or cells were processed in lysis buffer containing 1 × protease inhibitor cocktail followed by centrifugation to remove the lysate. p-STING, p-IRF3, p-TBK1and *β*-actin expression levels were analyzed by western blotting. An equal volume of the samples was loaded and separated by 10% sodium dodecyl sulfate PAGE (SDS-PAGE). The target bands were transferred to polyvinylidene difluoride (PVDF) membranes which were then blocked with 5% BSA and incubated with corresponding primary antibodies overnight at 4 °C. After that, the membranes were incubated with HPR-conjugated second antibodies (Proteintech, China). Pierce ECL Western blotting substrate was used to observe signals.

### 2.14 Quantitative reverse transcription PCR (qRT-PCR) analysis

RNA was isolated from cells or brain tissues with an RNA isolation kit (Vazyme, China) according to the manufacturer's specifications. Complementary DNA (cDNA) was synthesized using a PCR Thermal Cycler (Biorad, USA) with the HiScript cDNA kit (Vazyme, China). The qRT-PCR gene expression analysis was performed using ChamQ Universal SYBR qPCR Master Mix Kit (Vazyme, China) on Step One Plus (Applied Biosystems, Waltham, MA) with the appropriate primer pairs (Table S2). qRT-PCR data were normalized to GAPDH as housekeeping standard. Fold changes of target mRNAs were analyzed using the 2 ^−ΔΔCT^ method.

### 2.15 The subcutaneous and orthotopic glioma model

Male nude mice (20–22g) were subcutaneously implanted with 1 × 10^6^ GL261 cells in the right flank to establish a subcutaneous glioma model.

For the establishment of the orthotopic glioma model, male ICR or C57BL/6 mice (20–22g) were anesthetized using 2.0% isoflurane and positioned in a stereotactic instrument. The top of the animal's head was cleaned with 70% ethanol and betadine. A linear skin incision was made over the bregma, and 3% hydrogen peroxide was applied to the periost with a cotton swab. A 27G needle was used to drill a burr hole into the skull 0.5 mm anterior and 2 mm lateral to the bregma. A 10 μL gastight syringe (Hamilton) was then used to inject 5 μL of the Luc-GL261 cell suspension (1 × 10^6^ cells in PBS) in the striatum at a depth of 3.5 mm from the skull. The injection was done slowly over 10 min. The burr hole was occluded with glue to prevent leakage of cerebrospinal fluid, and the skin was closed with surgical sutures.

### 2.16 IVIS spectrum imaging analysis

Subcutaneous or orthotopic glioma-bearing mice were injected with Cy5.5-labeled HFn NPs or RGE-HFn NPs for evaluation of the biodistribution via the tail vein. At various time points, fluorescence signals were monitored via IVIS spectrum (PerkinElmer, USA). Tumor tissues and major organs including heart, liver, spleen, lung, and kidney were collected for *ex vivo* fluorescence examination using the same imaging system.

### 2.17 Evaluation of anti-glioma therapy and survival

The mice were monitored using an IVIS spectrum imaging system post-inoculation of orthotopic and luciferase-expressing glioma, and were randomly assigned into three groups on day 6. PBS, free SR717 or SR717@RGE-HFn (at a SR717 dose of 5 mg/kg) were intravenously administered to the tumor-bearing mice every third day for 15 days (five times in total). The survival and physical status of each animal were recorded for 30 days to plot the survival curve.

### 2.18 Flow cytometry analysis of tumor-associated immune cells

Brain tissues were harvested and single-cell suspensions were prepared by digestion with 0.25% trypsin followed by filtration through a 300-mesh sieve. The cells from each tumor were split into two 96-well plates and stained with a panel of T-cell antibodies and a panel of NK antibodies in parallel (Table S3). Flow cytometry was performed using the FACS Calibur flow cytometry system (BD Biosciences, CA) [43,44]. From each well, 50,000 events were recorded and analyzed with the Flowjo V10. The gating strategy for each specific population of immune cells is shown in Fig. S14.

### 2.19 Immunofluorescence analysis of tumor-associated immune cells

Tumor or brain tissues were harvested, fixed in paraformaldehyde, embedded within paraffin and sectioned. Tumor sections (10 μm) were deparaffinized and rehydrated. Sections were boiled in 10 mM citrate buffer (pH 6.0) at 95 °C for 15 min for antigen retrieval. Sections then were blocked with 5% goat serum in PBS for 1 h and incubated with anti-CD8 (1: 800) (Servicebio, China), anti-CD49b (1: 400) (Bioss, China), anti-CD86 (1: 500) (Bioss, China) and anti-TNF-*α* (1: 500) (Bioss, China) at 4 °C overnight, followed by incubation with Alex 488- or Cy5-conjugated goat-anti-rabbit IgG (1: 500) (Servicebio, China) at room temperature for 1 h. Cell nuclei were labeled with 4′,6-diamidino-2-phenylindole (DAPI). Images were captured using LSCM.

Optical imaging data were analyzed using ImageJ Fiji software32. Total cell numbers were calculated from manual counts of DAPI-labeled nuclei; counts were performed with the Fiji cell counter tool.

### 2.20 ELISA assay

Interleukin-2 (IL-2) in tumor samples was tested with a murine IL-2 ELISA kit (Biolegend, San Diego, CA) according to the manufacturer's instructions.

### 2.21 Statistical analysis

All data from at least three independent experiments were presented as mean ± standard deviation (SD). The differences between groups were analyzed using Student's *t*-test or one-way analysis of variance (ANOVA). In all cases, p-Values less than 0.05 were considered statistically significance (*p < 0.05, **p < 0.01, ***p < 0.001).

## 3 Results

### 3.1 Preparation and characterization of peptide-HFn NPs

We functionalized the HFn subunit with different tumor-targeting peptides, including RGE, Pep-1 and CGKRK peptides, at the N-terminus through a genetic engineering method to impart HFn NPs with tumor-homing and penetration properties. The peptide-fused proteins (peptide-HFn) were expressed in E. *coli* (BL21(DE3)) and purified using a previously described procedure [40] but with significant modifications and optimization in the construction and purification of expression vectors. We fused a 6 × His-tag to the C-terminus of HFn for purification. The fusion of biomacromolecules at the N-terminus of ferritin allows proteins or peptides to be displayed on the surface of HFn NPs assembled from the fused subunits [45]. 3D models of the yielded peptide-fused ferritin subunit and self-assembled peptide-HFn NPs were created using Modellar (v 9.2) simulation software, which showed that the fused peptide was located on the NP surface (Scheme 1). Western blot analysis indicated the successful expression of three peptide-HFn subunits, as shown in Fig. S1. The molecular weights of the peptide-HFn subunits were characterized to be in the range of 20–25 kDa according to the western blotting results. MALDI-TOF-MS was conducted to determine the molecular weight of RGE-HFn as 22.7 kDa, consistent with the theoretical molecular weight calculated based on its structure (Fig. S2).

As evidenced by DLS, HFn NPs exhibited a slight increase in average size after peptide modification while maintaining a narrow size distribution (Fig. S3), and the zeta potential of all nanoparticles was measured to be in the range of −6.97 mV to −9.45 mV (Table S1). Transmission electron microscopy (TEM) images showed the homogenous spherical cage-like structures of RGE-HFn, Pep-1-HFn, and CGKRK-HFn NPs, suggesting that the fusion proteins retained their unique assembly properties after peptide functionalization (Fig. S3).

### **3.2** In vitro assessment of peptide-HFn NPs for targeting glioma cells and traversing BBB

**Fig. 1:**
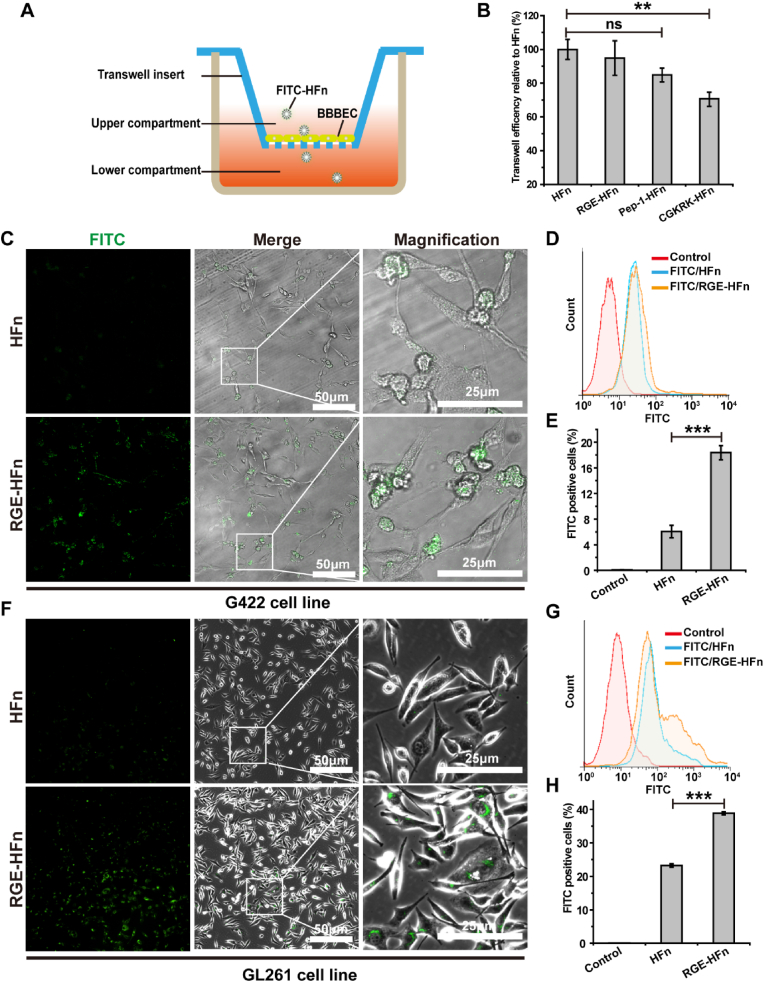
*In vitro* evaluation of peptide-HFn NPs for transcytosis and cell targeting capability. (A) Schematic illustration of an *in vitro* BBB model. (B) Quantitative analysis of transcytosis mediated by HFn and peptide-HFn NPs using the *in vitro* BBB model (*n* = 3 biologically independent samples per group). *p* > 0.05 means no statistical significance (ns) and *p < 0.05 compared to HFn NPs. (C and F) Confocal images of the G422 (C) or GL261 (F) glioma cells incubated with FITC-labeled HFn or RGE-HFn NPs for 4 h. (D and G) Representative flow cytometry histograms of G422 (D) or GL261 (G) cells incubated with FITC-labeled HFn and RGE-HFn NPs. (E and H) Quantitative analysis of the percentage of FITC positive G422 (E) or GL261 (H) glioma cells (*n* = 3 biologically independent samples per group). ***p < 0.001 compared to HFn NPs. Experiments were repeated twice independently with similar results. Significant differences were assessed using a one-way ANOVA with Tukey test (B, E and H). Data in (B, E and H) are presented as mean ± standard deviation (SD) from the second repeat.

We used a well-established murine brain microvascular endothelial (bEnd.3) cell monolayer as an *in vitro* BBB model to conduct a BBB transcytosis assay and investigate the effect of peptide functionalization on the ability of NPs to traverse the BBB. As shown in the native PAGE images presented in Fig. S4, the three peptide-HFn NPs maintained intact structures after traversing the BBB, which is essential for avoiding the undesirable leakage of payloads that may occur during the process of crossing the BBB [46]. Compared to naïve HFn NPs, whose ability to cross the BBB has been extensively studied both *in vitro* and *in vivo* [25], RGE-HFn NPs showed comparable penetration across the cell layer. Although Pep-1-HFn NPs showed slightly lower transcytosis efficiency than naïve HFn NPs, no significant difference was observed between them. The results indicated that functionalization with the RGE or Pep-1 peptide did not impair the transcytosis efficiency of HFn NPs (Fig. 1A and B). In contrast, the CGKRK peptide fusion protein exhibited a remarkable decrease in the ability to cross the BBB. We performed a fluorescence-based ELISA assay to investigate the affinity of different FITC-labeled NPs to recombinant murine TfR1. The ELISA result showed that peptide-HFn shared similar TfR1 binding affinity with HFn, indicating that the peptide fusion process did not influence the affinity of HFn to TfR1(Fig. S5).

The capability of peptide-HFn NPs to bind to living glioma cells was investigated by separately treating GL261 cells with FITC-labeled RGE-HFn, Pep-1-HFn, or CGKRK-HFn NPs. The cellular fluorescence intensities were measured using flow cytometry and normalized to that of the cells without any treatment, which were 28% for naïve HFn NPs, 42% for RGE-HFn NPs, 27% for Pep-1-HFn NPs, and 7% for CGKRK-HFn NPs. RGE-HFn exhibited the highest cellular internalization among the tested NPs (Fig. S6), which was most likely a result of the efficient binding of the RGE peptide to NRP-1 expressed on GL261 cell (Fig. S7). In contrast, the lowest cellular uptake was observed for CGKRK-HFn NPs, which was likely because the immobilization of the CGKRK peptide interfered with the interaction between the HFn subunit and TfR1 on glioma cells.

Laser scanning confocal microscopy (LSCM, Leica, USA) was used to visualize the cellular internalization of FITC-labeled RGE-HFn NPs in G422 and GL261 cells, and more NPs were observed in the cells treated with RGE-HFn NPs than in the cells treated with naïve HFn NPs, as evidenced by confocal images (Fig. 1C, F). This result was consistent with the flow cytometry analysis, which showed a stronger fluorescence signal in both cell lines treated with RGE-HFn NPs than in the cells treated with naïve HFn NPs (Fig. 1D–E, G-H). Based on the results described above, REG-HFn NPs not only exhibited an excellent capability of targeting glioma cells but also maintained their BBB-traversing property without destroying the original nanostructure of HFn NPs. As a result, RGE-HFn NPs were selected for subsequent *in vitro* and *in vivo* assays.

### **3.3** In vitro tumor penetration ability of RGE-HFn NPs

**Fig. 2:**
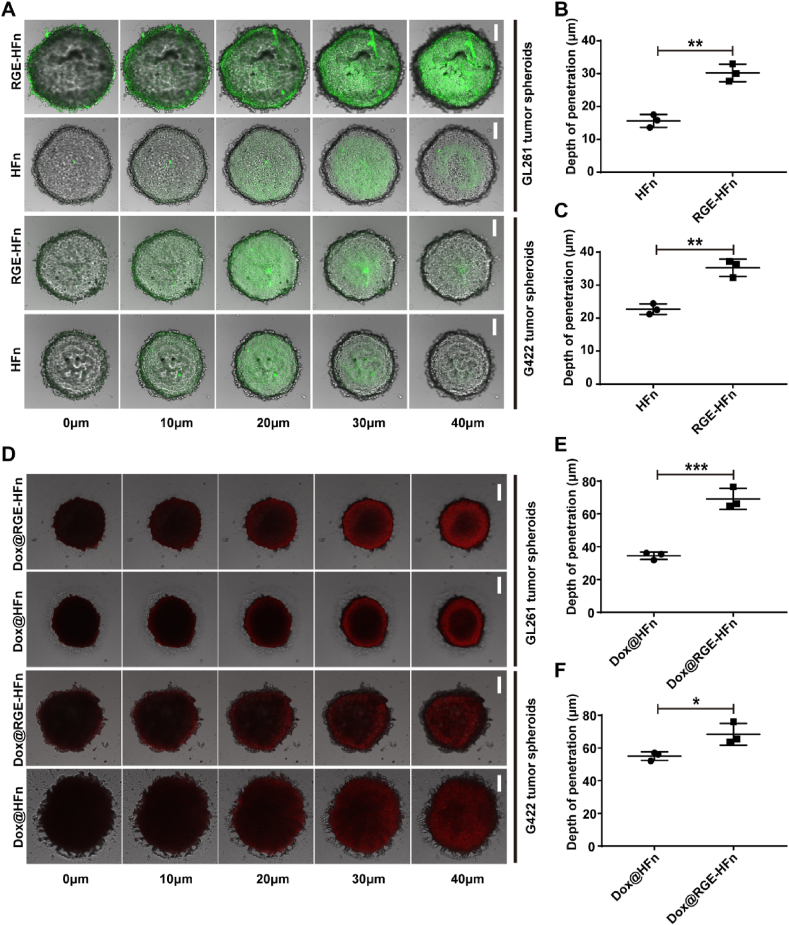
*In vitro* penetration of RGE-HFn NPs in 3D glioma spheroid models. (A) Penetration of FITC-labeled HFn and RGE-HFn NPs in GL261 (top) and G422 (bottom) glioma spheroids. (B and C) Quantitative analysis of the penetration depth of HFn and RGE-HFn NPs in GL261 (B) or G422 glioma spheroids (C) (*n* = 3 biologically independent samples per group). **p < 0.01 and ***p < 0.001 compared to HFn NPs. (D) Penetration of Dox@HFn and Dox@RGE-HFn NPs in G422 (top) and GL261 (bottom) glioma spheroids. (E and F) Quantitative analysis of the penetration depth of Dox@HFn and Dox@RGE-HFn NPs in GL261 (E) or G422 glioma spheroids (F) (*n* = 3 biologically independent samples per group). **p < 0.01 and ***p < 0.001 compared to Dox@HFn NPs. LSCM images were obtained from the top to the middle of the glioma spheroids in a Z-stack thickness of 10 μm. Scale bar, 50 μm. Experiments were repeated twice independently with similar results. Significant differences were assessed using a two-tailed unpaired Student's *t*-test (B, C, E and F). Data in (B, C, E and F) are presented as mean ± SD from the second repeat.

The targeting ability of nanocarriers not only contributes to cellular uptake by glioma cells but also enables the transport of therapeutic agents into deep tumor tissues. It has been shown that the RGERPPR motif could efficiently promote curcumin permeation into glioma tissue [47]. In addition to the cellular uptake assay, we further evaluated the penetration of RGE-HFn NPs using an *in vitro* 3D tumor spheroid transportation model that reflects many of the characteristics of solid tumors, including a dense and rigid extracellular matrix (ECM), heterogeneity, tight junctions between epithelial cells and high pressure [48,49]. FITC-labeled HFn or RGE-HFn NPs were incubated with G422 or GL261 tumor spheroid models, respectively, and the FITC fluorescence signal was monitored using LSCM. Z-stack images were obtained starting at the top of the spheroids in 10 mm intervals. As shown in Fig. 2A, the fluorescence signal of HFn NPs was distributed gradually from the surface to a depth of up to 20 μm within both tumor spheroid models, likely due to their small size. Intriguingly, tumor spheroids treated with RGE-HFn NPs exhibited much greater fluorescence intensity and a deeper penetration depth than HFn NPs (Fig. S8). A quantitative analysis of the penetration depth of NPs was conducted using the confocal software LAS AF (Leica, USA), indicating that RGE-HFn NPs penetrated much deeper to a distance of up to 30–40 μm in tumor spheroids than HFn NPs (Fig. 2B and C).

We subsequently investigated whether RGE-HFn NPs delivered cargos deep into the 3D tumor spheroids. We encapsulated Dox within the cavity of NPs as a model drug since it possesses a similar molecular weight as SR717 in addition to intrinsic fluorescence. We characterized the Dox content in HFn and RGE-HFn NPs. In an aqueous solution of Dox@HFn and Dox@RGE-HFn NPs, the concentration of HFn and RGE-HFn were 824.9 and 736.1 μg/mL respectively, as determined using a BCA protein assay, while that of Dox were measured to be 56.6 and 52.9 μg/mL respectively using an HPLC method. Accordingly, 58.8 and 54.5 Dox molecules were calculated to be encapsulated in an HFn NP and RGE-HFn NP respectively. Dox-loaded NPs were incubated with G422 or GL261 tumor spheroid models, and the fluorescence signal of Dox was monitored using LSCM. Fig. 2D illustrates that compared to HFn NPs, RGE-HFn NPs delivered more Dox into the middle of GL261 tumor spheroids. Additionally, Dox penetrated significantly deeper into both tumor spheroids when delivered in RGE-HFn NPs than in HFn NPs (Fig. 2E and F). Overall, HFn NPs with RGE peptide immobilization significantly improved the tumor penetration and uptake of encapsulated cargos.

### **3.4** In vivo tumor-targeting and BBB-crossing abilities of RGE-HFn NPs

**Fig. 3:**
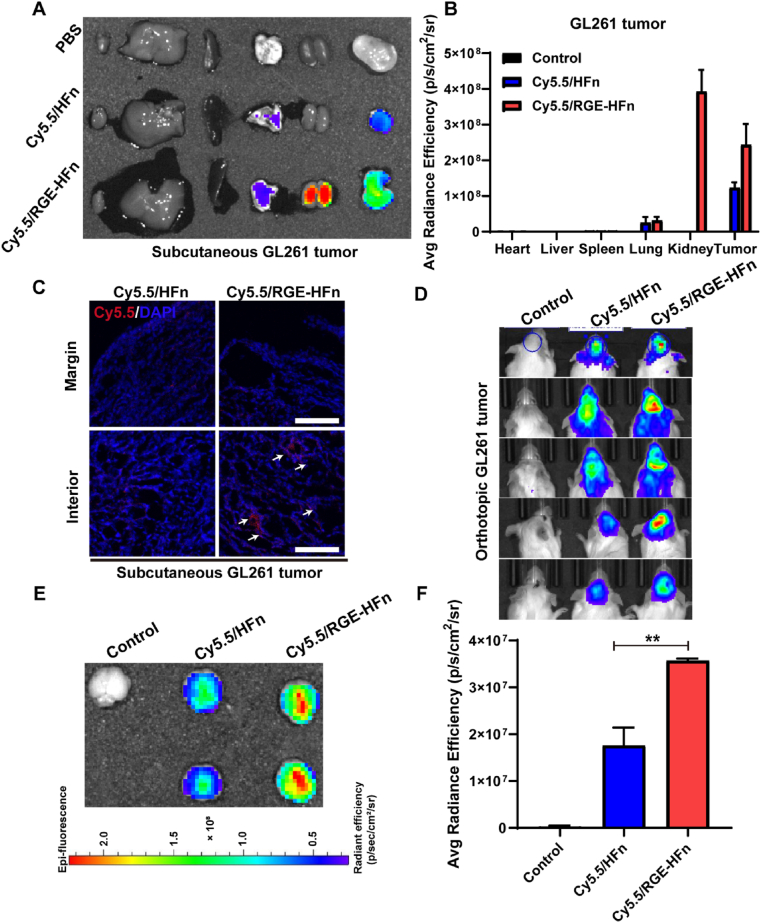
*In vivo* evaluation of RGE-HFn NPs for BBB crossing and glioma targeting capability. (A) Representative *ex vivo* images of subcutaneously xenografted GL261 tumor and organs that were excised at 24 h after intravenous injection of PBS, Cy5.5-labeled HFn or RGE-HFn NPs. (B) Fluorescence intensity of the excised tumors and organs was measured using an IVIS spectrum imaging system (*n* = 5 biologically independent mice per group). (C) Representative LSCM images of the excised tumor sections. White arrows indicate biodistribution of Cy5.5-labeled NPs. Scale bar, 50 μm. (*n* = 5 biologically independent mice per group). (D) Representative IVIS images of orthotopic GL261 glioma-bearing mice at 8 h after intravenous injection of PBS, Cy5.5-labeled HFn or RGE-HFn NPs. (*n* = 5 biologically independent mice per group). (E) Representative IVIS images of the brain tissues from the orthotopic GL261 glioma-bearing mice. (*n* = 5 biologically independent mice per group). (F) Fluorescence intensity of the excised brain tissues using IVIS spectrum imaging system (*n* = 5 biologically independent mice per group). **p < 0.01 compared to Cy5.5-labeled HFn NPs. Experiments were repeated twice independently with similar results. Significant differences were assessed using a one-way ANOVA with Tukey test (F). Data in (B and F) are presented as mean ± SD from the second repeat.

The *in vivo* tumor-targeting abilities of RGE-HFn NPs were evaluated using a mouse model in which GL261 glioma cells were grafted subcutaneously in the right flank. Mice were intravenously injected with Cy5.5-labeled HFn or RGE-HFn NPs 14 days after tumor inoculation. Compared to HFn NPs, significantly more RGE-HFn NPs accumulated in the subcutaneous GL261 tumor tissue, as confirmed by the IVIS imaging analysis of the Cy5.5 fluorescence signal (Fig. 3A and B). The fluorescence signals in the other major organs, including the heart, liver, spleen, lungs and kidneys, were also examined. Substantial amounts of RGE-HFn NPs were observed within the kidneys (Fig. 3A and B), which were quickly cleared in the next 48 h (data not shown). Tumor tissues were cryo-sectioned and the Cy5.5 fluorescence signal was visualized using LSCM. As shown in Fig. 3C, more RGE-HFn NPs diffused into the tumor tissue than HFn NPs. Taken together, engineering with the RGE peptide facilitated the enrichment of NPs in glioma tumor tissues.

We then constructed an orthotopic GL261 glioma model to assess the capability of NPs to traverse the BBB and target tumors. Tumor-bearing mice were intravenously injected with Cy5.5-labeled NPs on day 14 post-tumor inoculation and subjected to IVIS imaging analysis at 8 h following the injections. Higher accumulation of RGE-HFn NPs within the tumor region was observed compared to that of HFn NPs, suggesting the increased homing and retention of NPs in intracranial gliomas following functionalization with the RGE motif (Fig. 3D). Brains were subsequently isolated and imaged. As shown in Fig. 3E, stronger Cy5.5 signals were detected in the brain tissues treated with RGE-HFn NPs than in the brains treated with HFn NPs, which showed a relatively weaker fluorescence signal. Engineering with the RGE motif led to a significantly higher fluorescence intensity of NPs at the orthotopic glioma site, as further confirmed by a quantitative ROI analysis (Fig. 3F). More RGE-HFn NPs were observed in the deep region of glioma tissue compared to naïve HFn NPs, indicating tumor-homing activity of RGE engineered NPs (Fig. S9). Based on these results, RGE peptide functionalization remarkably improved the orthotopic glioma targeting efficiency of NPs without impairing their ability to traverse the BBB.

### **3.5** *Characterization of SR717@RGE-HFn NPs and pH-dependent release properties*

**Fig. 4:**
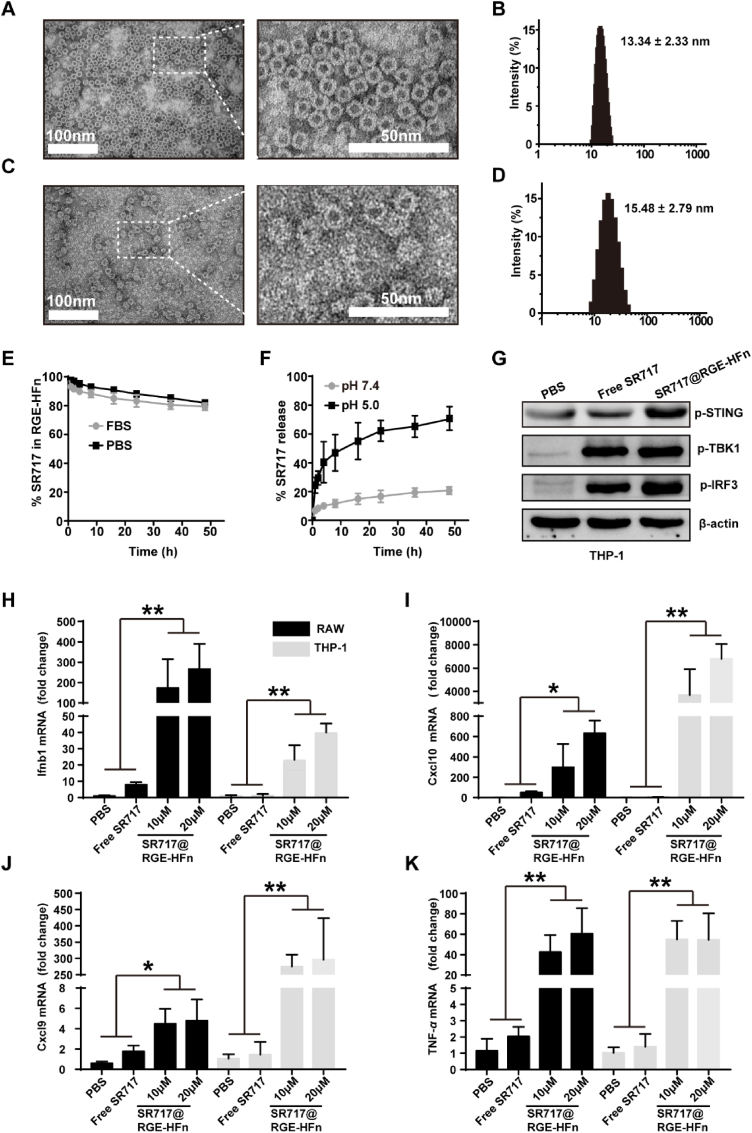
NP Characterization and *in vitro* evaluation of STING activation by SR717@RGE-HFn NPs. (A, C, B, D) Representative TEM images of RGE-HFn (A) and SR717@RGE-HFn NPs (C). DLS analysis of RGE-HFn (B) and SR717@RGE-HFn NPs (D). (E) SR717 release profile from SR717@RGE-HFn NPs in PBS with and without 10% FBS over 50 h (*n* = 3 biologically independent samples per group). (F) SR717 release profile from SR717@RGE-HFn NPs at pH 5.0 and pH 7.4 (*n* = 3 biologically independent samples per group). (G) Western blot analysis of p-STING, p-IRF3 and p-TBK1. *β*-actin is used as an internal reference. (H–K) qRT-PCR analysis of Ifnb1 (H), Cxcl10 (I), Cxcl9 (J) and TNF-*α* (K) mRNA expression in RAW and THP-1 cells after treatment with SR717@RGE-HFn NPs, free SR717 or PBS as control (*n* = 3 biologically independent samples per group). *p < 0.05 and **p < 0.01 compared to free SR717. Experiments were repeated twice independently with similar results. Significant differences were assessed using a one-way ANOVA with Tukey test (H–K). Data in (E–F) and (H–K) are presented as mean ± SD from the second repeat.

SR717, a potent non-nucleotide STING agonist, was loaded into the cavity of RGE-HFn NPs through a pH-mediated disassembly-reassembly procedure to obtain SR717@RGE-HFn NPs. The morphology of SR717@RGE-HFn NPs was visualized using TEM. Both RGE-HFn NPs (Fig. 4A) and SR717@RGE-HFn NPs (Fig. 4B) were monodispersed with a well-defined spherical morphology. TEM images also confirmed that SR717@RGE-HFn NPs maintained an intact structure after the disassembly and assembly processes. DLS results revealed that SR717@RGE-HFn NPs had a mean particle size of 15.48 nm with a narrow size distribution. The slight increase in particle size compared to RGE-HFn NPs (13.34 nm) was mostly a result of SR717 encapsulation [39,40] (Fig. 4B, D). The zeta potential of SR717@RGE-HFn NPs was almost identical to that of empty RGE-HFn NPs (Table S1). Thus, the encapsulation of SR717 had little effect on the physicochemical properties of RGE-HFn NPs. We further characterized the drug content in RGE-HFn NPs. In an aqueous solution of SR717@RGE-HFn NPs, the concentration of RGE-HFn was 723.1 μg/mL, as determined using a BCA protein assay, while that of SR717 was measured to be 44.6 μg/mL using an HPLC method. Accordingly, 88.6 SR717 molecules were calculated to be encapsulated in an RGE-HFn NP.

The stability of SR717@RGE-HFn NPs was evaluated by incubating SR717@RGE-HFn NPs in 10% FBS or PBS at 37 °C and monitoring the release profile of SR717 from NPs (Fig. 4E). No obvious drug release was detected in the presence of FBS during an incubation period of 50 h, which was similar to the release kinetics in PBS. This result implied that RGE-HFn NPs were stable under physiological conditions without the premature release of payloads before reaching the tumor site. HFn NPs have been reported to disassemble into protein subunits under acidic conditions to release payloads [26]. To verify the pH-dependent release of SR717 from RGE-HFn NPs, SR717@RGE-HFn NPs were incubated at 37 °C in PBS at pH 7.4 or 5.0, and SR717 release was monitored over time (Fig. 4F). SR717 was stably encapsulated in RGE-HFn NPs, with only 20% of SR717 released over 50 h when incubated under neutral conditions. In contrast, SR717 was released rapidly from RGE-HFn NPs with a half-life of merely 10 h when incubated at pH 5.0. A cumulative release of 65.6 ± 7% of SR717 was detected at pH 5.0, indicating that the RGE-HFn NPs were unstable at this pH and released SR717 in a pH-dependent manner.

### **3.6** *SR717@RGE-HFn NPs enhanced STING downstream signaling in vitro*

The effect of SR717@RGE-HFn NPs on the activation of the STING pathway was evaluated in a human monocyte cell line (THP-1). Western blot images showed that THP-1 cells treated with SR717@RGE-HFn NPs and free SR717 exhibited increased phosphorylation of STING (p-STING), IRF3 (p-IRF3) and TBK1 (p-TBK1), but PBS treatment did not result in increased p-STING, p-IRF3 or p-TBK1 levels (Fig. 4G). This result implied that SR717@RGE-HFn NPs successfully activated the STING pathway.

Interferon beta 1 (Ifnb1) is one of the main proteins expressed in response to activation of IRF3 and NF-κB, which are major signaling cascades triggered by STING activation [50,51]. In addition to IFN-*β*, C-X-C motif ligand 9 (Cxcl9) and C-X-C motif ligand 10 (Cxcl10) are two critical chemokines involved in downstream signaling of the STING pathway and are attributed to effector T cell recruitment [52], which is required for STING-dependent TNF-*α* production [53]. qRT-PCR analysis revealed that SR717@RGE-HFn NP treatment markedly elevated the mRNA levels of Ifnb1, Cxcl10, Cxcl9, and TNF-*α* compared with free SR717 in both RAW and THP-1 cells (Fig. 4H–K). Induction of the expression of these mRNAs was not detected in cells treated with blank RGE-HFn NPs (Fig. S10). The results verified that the upregulation of gene expression downstream of STING signaling was induced by SR717, which was more potent in activating STING pathway signaling when encapsulated within RGE-HFn NPs. The increase in STING activation is likely due to the higher stability and longer half-life of SR717 in the NP form, and the NP-mediated enhancement is dose-dependent, as shown in Fig. 4H–K. More importantly, no obvious cytotoxicity was observed in RAW, THP-1 cells or bone marrow-derived monocytes (BMDMs) treated with either free SR717 or SR717@RGE-HFn NPs at all tested concentrations, revealing the biocompatibility of SR717@RGE-HFn NPs (Fig. S11). Overall, RGE-HFn NPs improved the potency of SR717 for inducing proinflammatory cytokine cascades in monocyte and macrophage cell lines in a safe and dose-dependent manner.

### **3.7** *SR717@RGE-HFn NPs improved anti-tumor activity and prolonged survival*

**Fig. 5:**
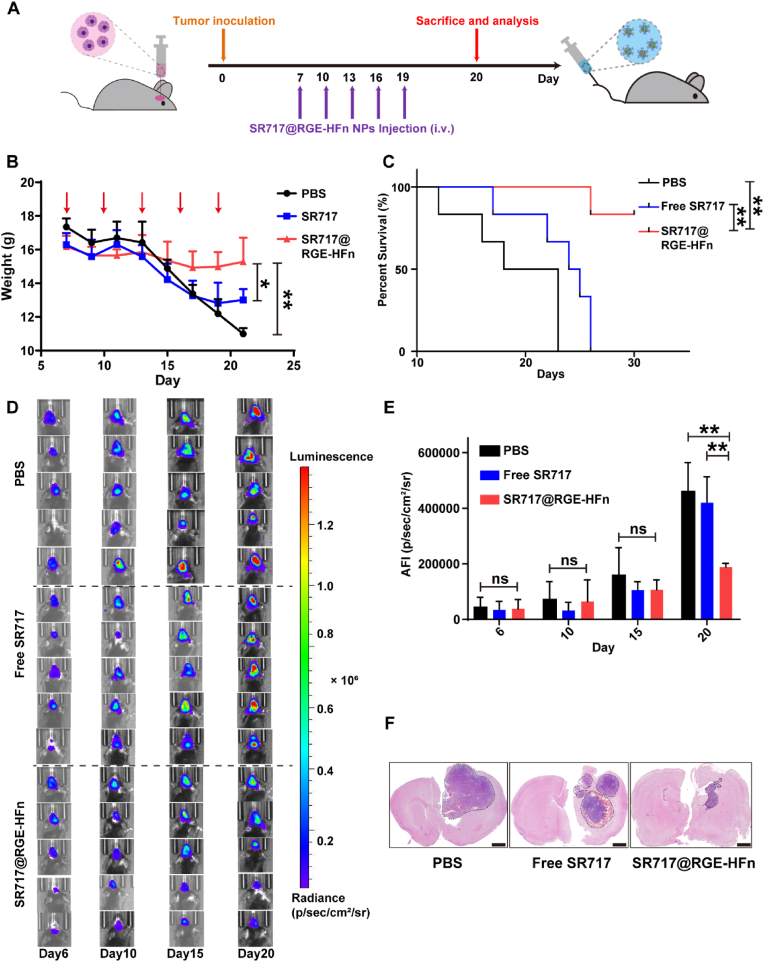
*In vivo* evaluation of anti-glioma activity of SR717@RGE-HFn NPs. (A) Schematic illustration of the treatment regimen. (B) Body weight changes after intravenous administration of the indicated treatment groups (*n* = 6 biologically independent mice per group). *p < 0.05 and **p < 0.01 compared to PBS or free SR717. Red arrows indicate the time points for intravenous injections during the study. (C) The survival curve of mice treated with the indicated formulations (*n* = 6 biologically independent mice per group). **p < 0.01 compared to PBS or free SR717. (D) Bioluminescence signal change correlating to tumor growth over time following inoculation (*n* = 5 biologically independent mice per group); the bar indicates radiant efficiency from 9.5 × 10^3^ to 1.3 × 10^6^. (E) Semi-quantification of the tumor bioluminescence signal (*n* = 5 biologically independent mice per group). **p < 0.01 compared to PBS or free SR717. (F) H&E staining of brain tissues. Scale bar, 1 mm. Experiments were repeated twice independently with similar results. Significant differences were assessed using a one-way ANOVA with Tukey test (B and E). Statistical significance was calculated by log-rank test (C). Data in (B and E) are presented as mean ± SD from the second repeat.

The anti-glioma efficacy was evaluated in orthotopic luciferase-expressing GL261 tumor-bearing C57BL/6 mice after the intravenous administration of different treatments according to the regimen, as shown in Fig. 5A. We first monitored the body weights of mice from different treatment groups. Compared with the other treatment groups, systemic delivery of SR717@RGE-HFn NPs resulted in delayed body weight loss (Fig. 5B) and significantly prolonged animal survival with an improved durable cure rate (83%) (Fig. 5C). Meanwhile, free SR717 treatment improved the physical status and prolonged the median survival time of the animals, with a slower drop in body weight than the PBS-treated control group, suggesting the modest therapeutic effect of free SR717. An IVIS Spectrum *in vivo* imaging system was used to monitor the growth of glioma inside the mouse brain, as shown in Fig. 5D. The results showed rapid tumor growth in the PBS-treated control group, while moderate inhibition of tumor growth was observed in mice treated with free SR717 during the first two weeks. Notably, SR717@RGE-HFn NPs exhibited the strongest anti-glioma effect, with a 55.3% reduction in tumor volume compared to PBS-treated animals (Fig. 5E). On day 20 post-inoculation, brain tissues were isolated and subjected to H&E staining, revealing a significant reduction in the tumor area after SR717@RGE-HFn NP treatment (Fig. 5F). Together, these results indicated that RGE-HFn NPs remarkably improved the anti-glioma efficacy of SR717, which was most likely due to the combined functions of BBB crossing and glioma homing.

### 3.8 *SR717@RGE-HFn NPs stimulated a potent anti-tumor immune response* via *the STING pathway*

**Fig. 6:**
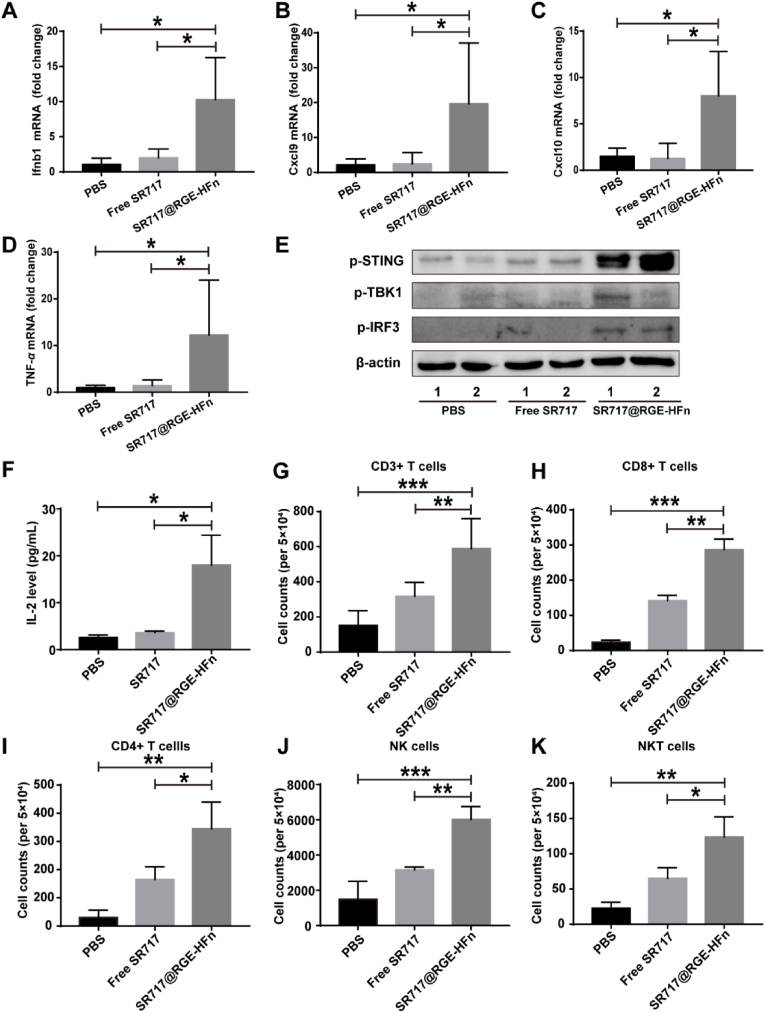
*In vivo* anti-glioma immune response triggered by SR717@RGE-HFn NPs. (A–D) qRT-PCR analysis of Ifnb1 (A), Cxcl9 (B), Cxcl10 (C) and TNF-*α* (D) mRNA expression in orthotopic gliomas after intravenous administration of the indicated treatment groups (*n* = 5 biologically independent mice per group). *p < 0.05 compared to PBS or free SR717. (E) Western blot analysis of expression levels of p-STING, p-TBK1 and p-IRF3 in orthotopic glioma tissues. *β*-actin is used as an internal reference. (F) ELISA analysis of IL-2 expression in orthotopic glioma tissues (*n* = 5 biologically independent mice per group). *p < 0.05 compared to PBS or free SR717. (G–K) Flow cytometry analysis of CD3^+^ T cells (G), CD3^+^CD8^+^ T effector cells (H), CD3^+^CD4^+^ T helper cells (I), CD3^−^NK1.1^+^ NK cells (J) and CD3^+^NK1.1^+^ NKT cells (K) in glioma tissues after treatment with the indicated formulations. Approximately 50,000 events/sample were recorded and analyzed with FlowJo V10 (*n* = 5 biologically independent mice per group). *p < 0.05, **p < 0.01 and ***p < 0.001 compared to PBS or free SR717. Experiments were repeated twice independently with similar results. Significant differences were assessed using a one-way ANOVA with Tukey test (A-D and F–K). Data in (A–D) and (F–K) are presented as mean ± SD from the second repeat.

We investigated the composition of the glioma TME at the end of the treatment regimen, including immune cells, proteins and cytokines, to better understand the therapeutic mechanism of SR717@RGE-HFn NPs in a mouse model of orthotopic glioma. Levels of the Ifnb1, Cxcl9, Cxcl10 and TNF-*α* mRNAs in glioma tissues from different treatment groups were determined using qRT-PCR to evaluate the activation of STING signaling. SR717@RGE-HFn NP treatment induced superior immunostimulatory activity, as evidenced by the significantly improved mRNA expression of Ifnb1 (2–3-fold), Cxcl9 (4-fold), Cxcl10 (3-fold), and TNF-*α* (4–5-fold) compared to the PBS and free SR717 groups (Fig. 6A–D).

We then investigated the protein expression of p-STING, p-IRF3 and p-TBK1 in tumor tissues from different treatment groups. Western blot analysis revealed higher levels of p-STING, p-IRF3 and p-TBK1 in the tumors treated with SR717@RGE-HFn NPs, but negligible levels were detected in PBS- and free SR717-treated tumors (Fig. 6E). Based on these results, SR717@RGE-HFn NPs successfully activated the STING pathway in the glioma TME. The level of IL-2 protein, an important indicator of T cell activity and proliferation, was assessed using ELISA. Compared with the free SR717 and PBS groups, IL-2 levels in the SR717@RGE-HFn NP treatment group were increased by 4.96- and 6.97-fold, respectively, suggesting that SR717 substantially improved the adaptive T cell response within the glioma TME via the STING pathway when delivered by RGE-HFn NPs (Fig. 6F).

Activation of the STING pathway in the TME has been shown to promote the infiltration of lymphocytes, the major mediator of effective cancer immunotherapy [54]. We subsequently evaluated the effect of SR717@RGE-HFn NPs on immune cell infiltration within the glioma TME using flow cytometry 24 h after the final injection of different formulations. Compared to the PBS and free SR717 groups, the SR717@RGE-HFn NP group exhibited 4.57- and 1.94-fold increases in the number of tumor-infiltrating CD3^+^ T cells and 2.02- and 9.56-fold increases in the number of CD3^+^CD8^+^ T effector cells, respectively (Fig. 6G and H). Increased recruitment of tumor-infiltrating CD3^+^CD4^+^ T helper cells was also observed following SR717@RGE-HFn NP treatment compared with the other treatments (Fig. 6I). NK cells, known as “tumor killers”, are another important group of cytotoxic lymphocytes; these cells participate in the innate immune response and promote the adaptive immune response [55]. After treatment with SR717@RGE-HFn NPs, the number of NK cells was increased by 1.91- and 4.04-fold compared to the levels observed in the free SR717 and PBS groups, respectively (Fig. 6I). Treatment with SR717@RGE-HFn NPs also resulted in the increased infiltration of NK T cells compared to the free SR717 and PBS groups (Fig. 6J). Overall, SR717@RGE-HFn NPs elicited a strong anti-glioma immune response by recruiting immune cells, including cytotoxic T lymphocytes (CTLs), DCs, and NK cells, to enhance antitumor efficacy through a mechanism that was probably dominated by the STING pathway.

**Fig. 7:**
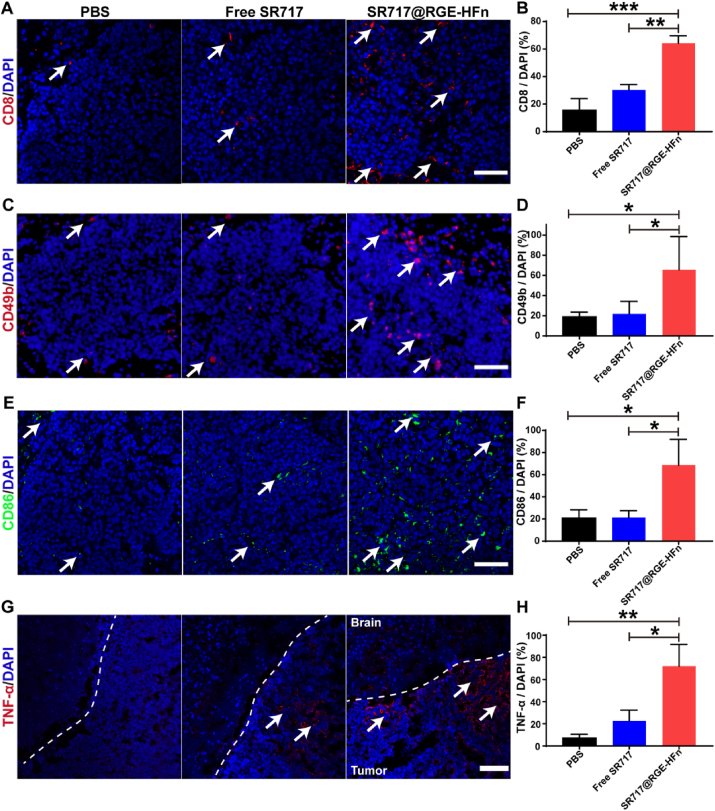
Increased infiltration of CTLs, DCs, NKs and elevated expression of TNF-*α* in glioma TME after treatment with SR717@RGE-HFn NPs. (A, C, E) Representative immunofluorescence images of tumor sections for glioma-infiltrating CD8^+^ T cells (A), CD49b^+^ NK cells (C) and CD86^+^ DCs (E). (*n* = 5 biologically independent mice per group). Scale bar, 50 μm. (G) Immunofluorescence staining of TNF-*α* in tumor tissues after intravenous injection of the indicated groups. Scale bar, 100 μm. White arrows highlight the indicated immune cells and TNF-*α* in tumor sections. (B, D, F, H) Treatment withSR717@RGE-HFn NPs resulted in a significant increase of CD8^+^ T cells (B), CD49b^+^ NK cells (D), CD86^+^ DCs (F) and TNF-*α* production (H) in tumor tissues (*n* = 5 biologically independent mice per group). *p < 0.05, **p < 0.01 and ***p < 0.001 compared to PBS or free SR717. Experiments were repeated twice independently with similar results. Significant differences were assessed using a one-way ANOVA with Tukey test (B, D, F and H). Data in (B, D, F and H) are presented as mean ± SD from the second repeat.

To further study the local immune response, brain tissues were collected and cryo-sectioned on day 20 after tumor inoculation for immunofluorescence staining analysis. As shown in Fig. 7A and B, the total number of tumor-infiltrating CD8^+^ T cells increased significantly following treatment with SR717@RGE-HFn NPs, while the numbers of CD8^+^ T cells in the PBS- and free SR717-treated groups were fairly similar and remained at a low level. In addition, treatment with SR717@RGE-HFn NPs improved the infiltration of NK cells (CD49b^+^ cells) into the glioma TME by 2.8-fold compared with free SR717 treatment (Fig. 7C and D). We next evaluated the presence of DCs in gliomas by performing immunofluorescence staining for CD86, a maturation marker overexpressed on activated tumor-infiltrating DCs [56]. As shown in Fig. 7E and F, the surface expression of CD86 was upregulated by 3.2-fold in the SR717@RGE-HFn NP-treated group compared with the free SR717-treated group, indicating that SR717@RGE-HFn NPs promoted an influx of DCs to create an innate inflammatory niche with the potential to prime adaptive immunity [57]. Furthermore, activation of the STING pathway also triggers proinflammatory responses through the NF-κB pathway [58], leading to the production of inflammatory cytokines, especially TNF-*α* [59]. The TNF-*α* protein level (Fig. 7G and H) was elevated by 3.18- and 8.75-fold in the glioma TME after treatment with SR717@RGE-HFn NPs compared to the free SR717 or PBS groups, respectively. The enhanced production of TNF-*α* in the glioma TME suggested that SR717@RGE-HFn NPs stimulated a profound anti-glioma immune response. Taken together, RGE-HFn NPs effectively delivered SR717 to orthotopic glioma, activated the STING pathway locally, induced widespread immune cell infiltration, and elicited a potent antitumor response.

### **3.9** *Hematological and histological analyses*

The possible adverse effects of SR717@RGE-HFn NPs were investigated through hematological assessment and histological examination. Serum levels of biochemical indicators, including alanine aminotransferase (ALA), aspartate transaminase (AST) and blood urea nitrogen (BUN), were detected to assess liver and kidney functions. The levels of these indicators in mice treated with SR717@RGE-HFn NPs were all within the reference ranges and comparable to the PBS and placebo control groups (Fig. S12), suggesting that NP treatment induced no obvious hepatic or renal toxicity. Moreover, H&E staining did not show pathological changes in major organs, including the heart, liver, spleen, lung, kidney and brain among the treatment groups (Fig. S13). These results confirmed the excellent biocompatibility of the developed SR717@RGE-HFn NPs, suggesting that they represent promising therapeutic candidates for orthotopic glioma treatment.

## 4 Discussion

Numerous researchers have attempted to overcome the BBB and improve drug delivery efficiency into the brain. The ability of therapeutics to traverse the BBB might be substantially increased via a transcytosis-mediated mechanism that targets TfR1 on cerebrovascular endothelial cells [25]. Other studies have shown that several peptide motifs have potential as targeting ligands for glioma cells. In the present study, HFn NPs that traverse the BBB through TfR1-mediated transcytosis were engineered to display targeting peptides on their surface without generating any toxic byproducts that are usually involved in chemical functionalization. With dual-targeting properties, the yielded RGE-HFn NPs penetrated deep into the glioma tissue and delivered STING agonists once past the BBB. Additionally, as a natural carrier existing in mammals [24], HFn-based NPs were safer than artificial liposomes or polymeric nanocarriers that might introduce toxic compounds during preparation and degradation processes. Our study showed that the intravenous administration of RGE-HFn NPs did not exert any obvious adverse effects on blood biochemical indicators or pathology of major organs, indicating the excellent biocompatibility of the peptide-HFn delivery system.

Glioma-associated immunosuppression has been known for decades [12]. The intra-tumoral administration of cyclic dinucleotide-based STING agonists improves the therapeutic outcomes of glioma in both mouse and dog models, a result of enhanced type-I IFN, CXCL10 and CCL5 signaling and T cell infiltration into the brain and conversion of the glioma-associated immunosuppressive TME to an immunogenic TME [60,61]. Although STING agonists have shown promising results for glioma treatment, most of them are usually administered through the intra-tumoral route due to their systemic toxicity and poor pharmacokinetic and physicochemical properties, which significantly limit their potential for clinical translation [37,62]. Despite the substantial achievements in BBB drug delivery technology, the development of strategies to achieve brain delivery of STING agonists has experienced very limited success.

Here, we described a proof-of-concept for the design and preclinical use of peptide-engineered HFn NPs to deliver a STING agonist to intracranial glioma and stimulate a potent anti-glioma immune response. Three peptides, RGE, Pep-1 and CGKRK, were fused to the N-terminus of the HFn subunit through genetic engineering to construct three functionalized HFn NPs. Among them, RGE-HFn NPs showed the highest glioma cellular internalization while maintaining BBB-traversing properties, suggesting that the immobilization of RGE peptide on the surface of HFn NPs improved the glioma-homing ability without interfering with TfR1-mediated BBB transcytosis. The RGE peptide (RGERPPR) is a tumor-penetrating peptide that possesses a high binding affinity for NRP-1, a transmembrane glycoprotein that is overexpressed on glioma cells [28]. According to many studies, RGE peptide not only enhances the delivery of therapeutics to tumors but also facilitates drug distribution throughout the whole of tumor tissues instead of only tumor cells which are alongside tumor vessels [27]. As expected, LSCM images of 3D tumor spheroid models showed greater accumulation and deeper penetration of RGE-HFn NPs in glioma spheroids.

We then investigated the biodistribution of intravenously injected RGE-HFn NPs in a subcutaneous tumor-bearing mouse model and an orthotopic tumor-bearing mouse model. IVIS imaging analysis showed that the accumulation of RGE-HFn NPs in tumor tissues was approximately two-fold higher than that of naïve HFn NPs following intravenous administration. LSCM images of tumor cryosections further confirmed that RGE functionalization significantly improved NP diffusion in tumor tissues, which was often required for successful tumor therapy.

A non-nucleotide STING agonist, SR717, was effectively encapsulated within RGE-HFn NPs through electrostatic interactions. RGE-HFn NPs possess a unique pH-dependent drug release profile, which is essential for site-specific drug delivery, preventing the premature leakage of therapeutics during systemic circulation and rapid release of therapeutics upon arrival at the intracranial glioma. Based on the aforementioned results, RGE-HFn NPs were considered a promising drug delivery system that targeted and penetrated glioma upon traversing the BBB, and they were subsequently evaluated for anti-glioma efficacy in an orthotopic glioma mouse model. Intravenously administered SR717@RGE-HFn NPs resulted in improved physical status, prolonged survival of glioma-bearing mice and delayed growth of orthotopic glioma compared with free SR717, most likely due to the increased accumulation of SR717 within tumor tissues when delivered by RGE-HFn NPs. Thus, RGE-HFn NPs provided multiple benefits in treating orthotopic glioma by improving tumor-homing and BBB-crossing ability.

We further investigated whether RGE-HFn NPs induced a potent innate immune response in glioma via the STING pathway. Western blotting and qRT-PCR were performed and showed that treatment with SR717@RHE-HFn NPs upregulated the expression of STING downstream effectors and increased the mRNA levels of Ifnb1, Cxcl9, Cxcl10 and TNF-*α*, suggesting successful activation of the STING pathway by SR717 released under acidic conditions within glioma tissue. Additionally, increased infiltration of activated T cells, NK cells and DCs was observed within glioma tissues using flow cytometry and immunofluorescence staining, indicating the increased recruitment of immune cells into the glioma TME.

The lack of an effective brain delivery strategy has limited the efficacy of immunotherapy for brain tumors. In addition, a balance between the antitumor efficacy and immunotoxicity of STING agonists following systemic administration is particularly difficult to achieve. Thus, it is challenging to elicit efficient anti-glioma immune responses within the brain. In the present study, HFn NPs that have intrinsic BBB-crossing ability were engineered with a tumor penetration peptide RGE, termed RGE-HFn NPs, to facilitate delivery of the STING agonist SR717 into the brain tumor. *In vitro* and *in vivo* studies revealed that intravenously injected SR717@RHE-HFn NPs triggered a potent glioma-specific innate immune response and remarkably delayed the growth of orthotopic glioma without exhibiting apparent systemic toxicity. The anti-glioma effects of SR717@RGE-HFn NPs might be further improved by administration in combination with synergistic therapeutics (e.g., chemotherapeutics, immune checkpoint inhibitors and cytokines) or treatment modalities (e.g., radiation), which should provide additional benefits in addition to those of SR717@RGE-HFn NPs alone. Future investigations of local immunological memory and long-lasting systemic antitumor immunity will be conducted to fully elucidate whether SR717@RHE-HFn NPs prevent glioma recurrence and metastases. Collectively, peptide-fused protein NPs represent a simple and versatile brain delivery platform that may tremendously expand the scope of STING agonist-based cancer therapy.

## 5 Conclusion

In summary, we developed a new drug delivery platform with dual-targeting potential that enabled the homing of systemically administered STING agonists to the brain for glioma immunotherapy. This study provided a proof-of-concept that intravenous injection of SR717@RGE-HFn NPs effectively activates the STING pathway and exerts immunoregulatory effects within the intracranial glioma TME, leading to the inhibition of tumor progression with excellent biocompatibility. The developed RGE-HFn NP platform exhibits great potential for glioma treatment by targeting the STING pathway and shows enormous promise to solve the major challenges associated with CNS-directed drug delivery.

## CRediT authorship contribution statement

**Bin Wang:** Investigation, Methodology, Data curation, Writing – original draft. **Maoping Tang:** Methodology, Writing – review & editing. **Ziwei Yuan:** Investigation, Methodology. **Zhongyu Li:** Writing – review & editing. **Bin Hu:** Writing – review & editing. **Xin Bai:** Methodology. **Jinxian Chu:** Methodology. **Xiaoyang Xu:** Methodology, Supervision, Writing – review & editing. **Xue-Qing Zhang:** Conceptualization, Supervision, Funding acquisition, Writing – review & editing.

## Declaration of competing interest

The authors declare that they have no known competing financial interests or personal relationships that could have appeared to influence the work reported in this paper.
