# Supplementary material for: Targeted delivery of a STING agonist to brain tumors using bioengineered protein nanoparticles for enhanced immunotherapy
Source: Bioact Mater. 2022 Mar 1;16:232–48. doi: 10.1016/j.bioactmat.2022.02.026 (PMC8965725; doi:10.1016/j.bioactmat.2022.02.026)
Supplement: Multimedia component 1 [file mmc1.docx]

Appendix A. Supplementary data for

**Targeted delivery of a STING agonist in brain tumor with enhanced immunotherapy using bioengineered protein nanoparticles**

Bin Wang, Maoping Tang, Ziwei Yuan, Zhongyu Li, Bin Hu, Xin Bai, Jinxian Chu, Xiaoyang Xu^**^, Xue-Qing Zhang^*^

*Corresponding author. Email: xueqingzhang@sjtu.edu.cn

**Corresponding author. Email: xiaoyang@njit.edu

**This PDF file includes:**

Figs. S1 to S14

Tables S1 to S3


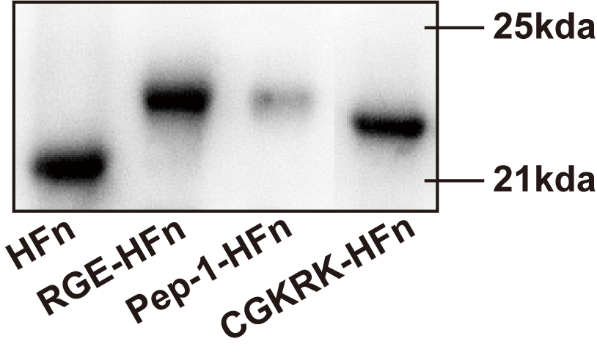


**Fig. S1.** The expression of HFn, RGE-HFn, Pep-1-HFn and CGKRK-HFn subunits were detected by western blot analysis.


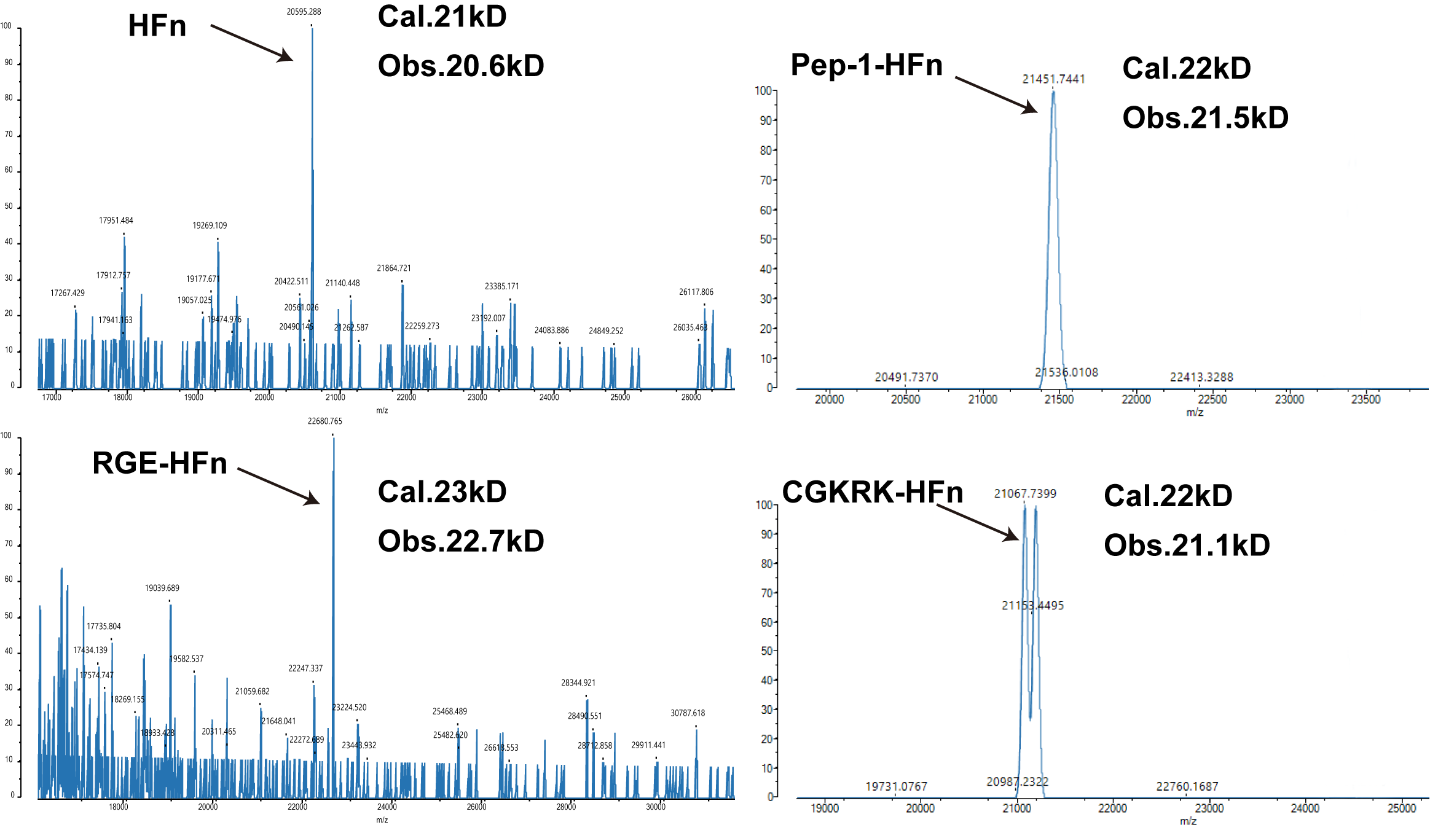


Fig. S2. MALDI-TOF-MS characterization of HFn, RGE-HFn, Pep-1-HFn and CGKRK-HFn subunits.


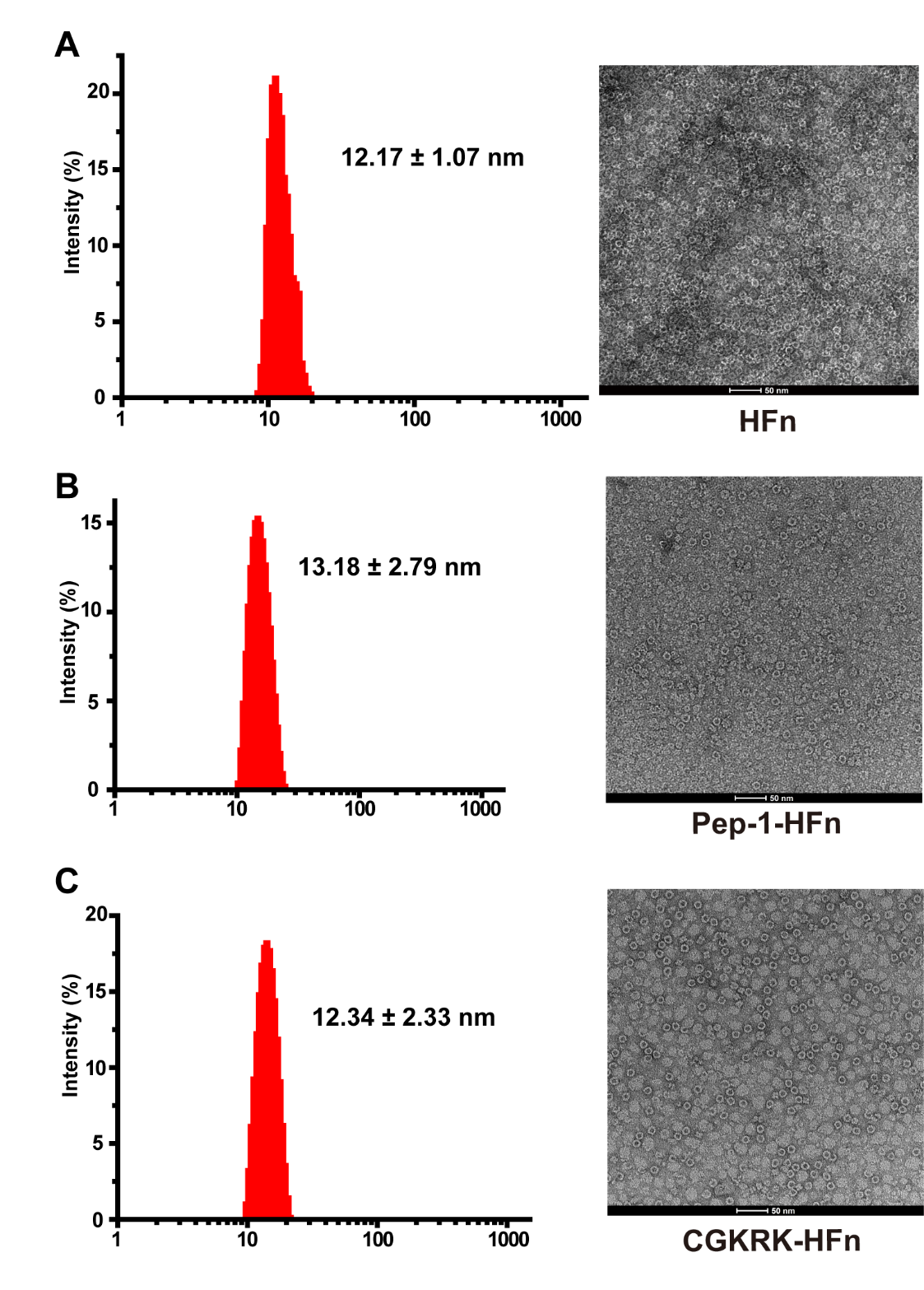


Fig. S3. Characterization of HFn, Pep-1-HFn and CGKRK-HFn NPs. (A-C) DLS (left) and TEM analysis (right) of HFn (A), Pep-1-HFn (B) and CGKRK-HFn (C) NPs.


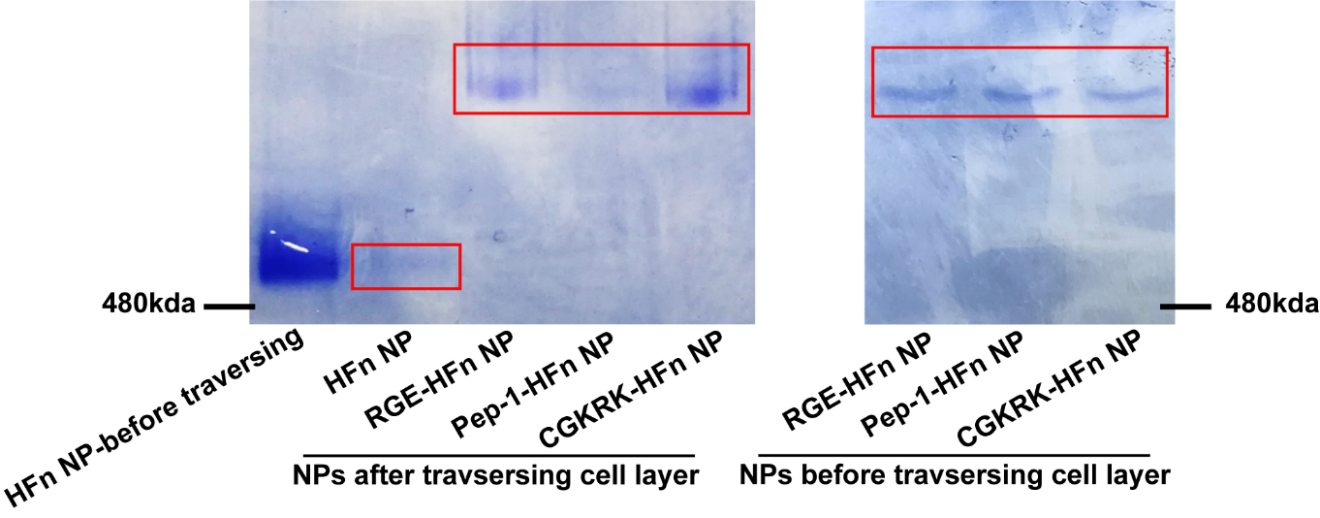


Fig. S4. Native PAGE analysis of the HFn NPs (fused and non-fused) before or after traversing the in vitro BBB model.


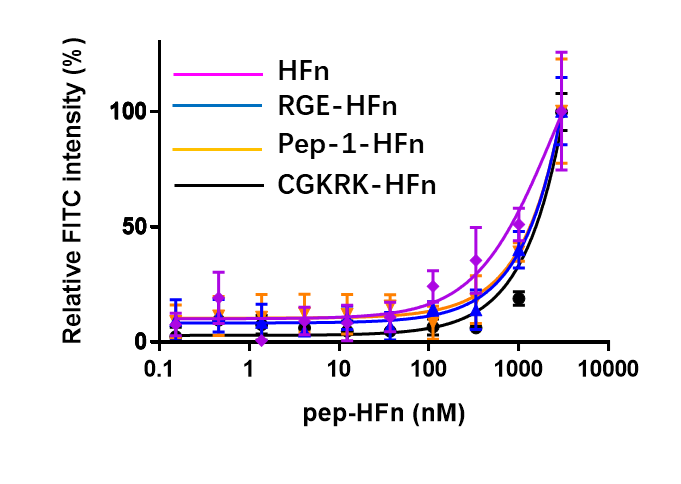


Fig. S5. Binding affinity of FITC-labeled HFn or peptide-HFn NPs to recombinant murine TfR1 characterized by fluorescence-based ELISA (*n* = 3 biologically independent samples per group). Experiments were repeated twice independently with similar results. Data are presented as mean ± SD from the second repeat.


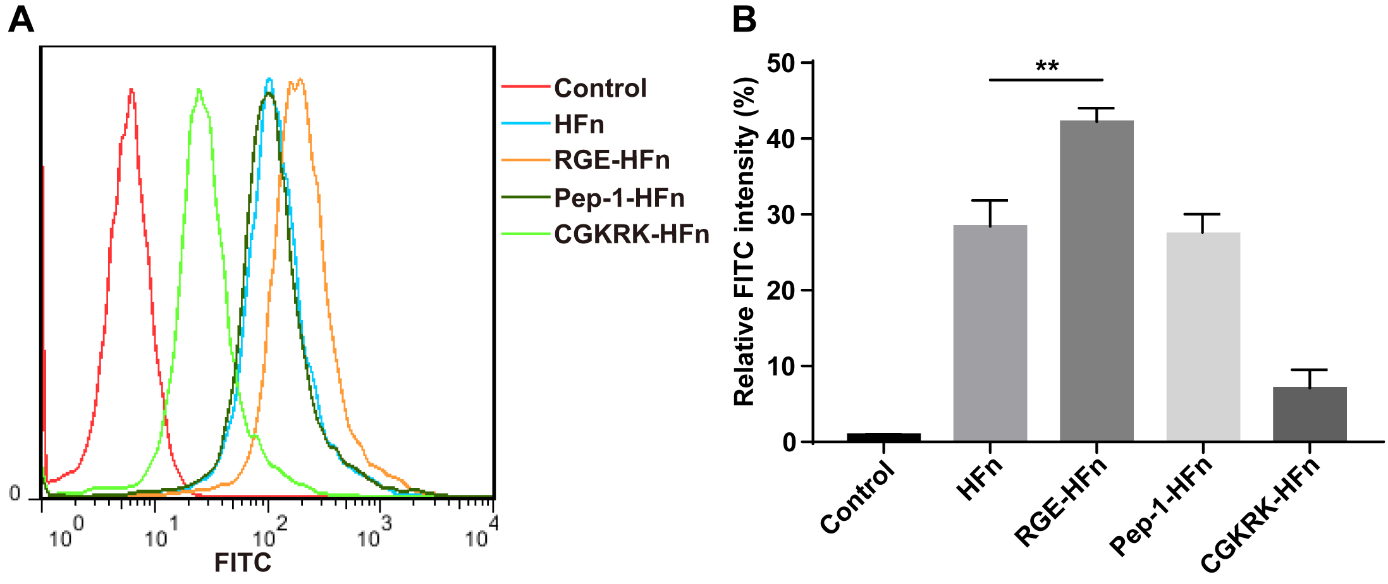


Fig. S6. Glioma targeting ability of HFn and peptide-HFn NPs. (A) Flow cytometry analysis of Gl261 glioma cells treated with HFn or peptide-HFn NPs. (B) Quantitative analysis of the relative fluorescence intensity for each treatment group, which were normalized against the control group (untreated cells) in panel A (*n* = 3 biologically independent samples per group). ***p* < 0.01 compared to HFn NPs. Experiments were repeated twice independently with similar results. Significant differences were assessed using a one-way ANOVA with Tukey test (B). Data in (B) are presented as mean ± standard deviation (SD) from the second repeat.


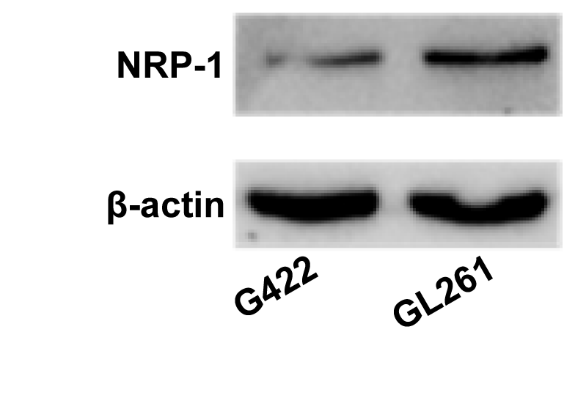


**Fig. S7.** Western blot analysis of NRP-1 expression in G422 and GL261 cells. *β*-actin is used as an internal reference.


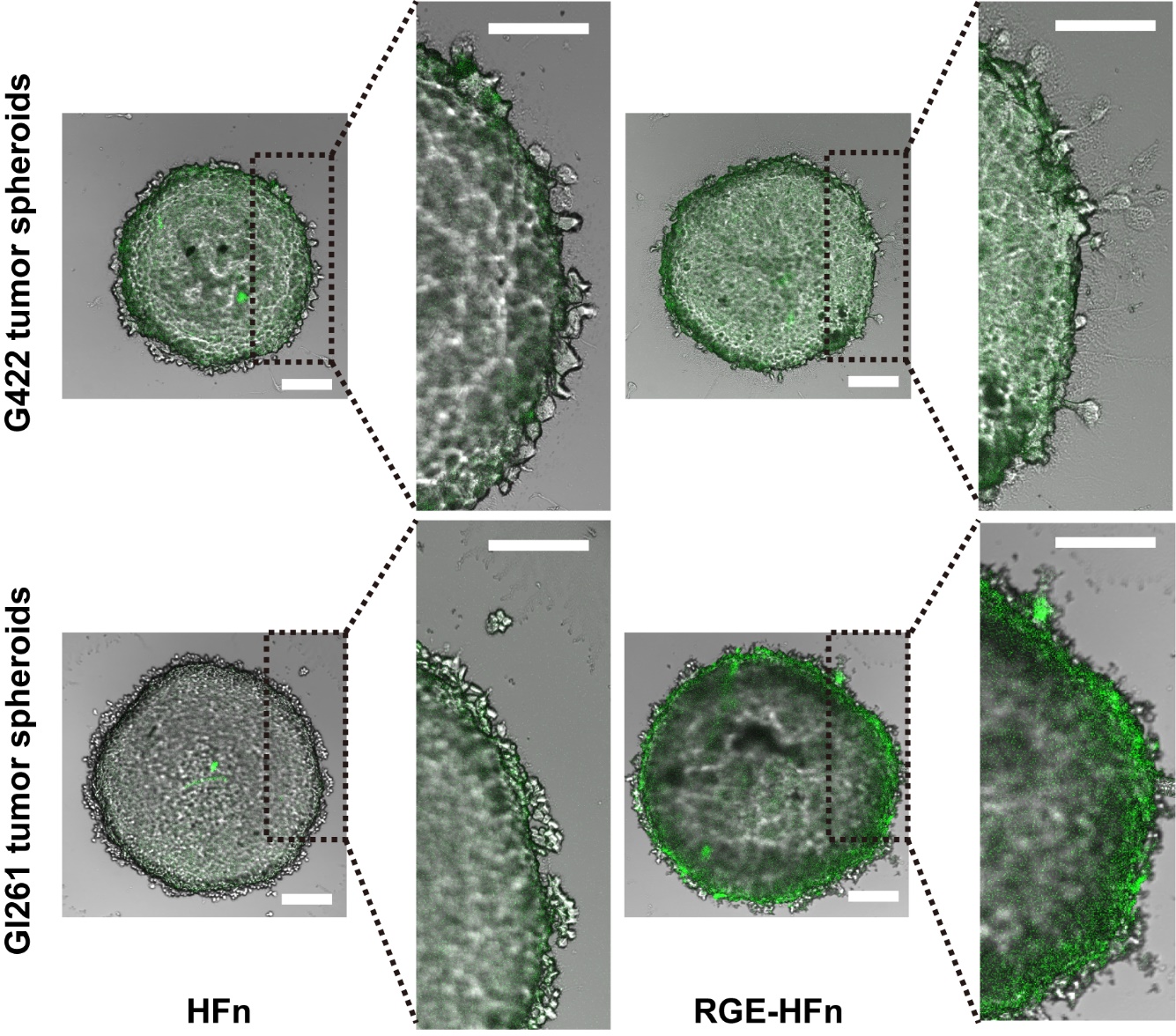


Fig. S8. Z-stack images were obtained starting at the top of the spheroids in 10 μm intervals. Magnified confocal images at the depth of 10 μm demonstrated that RGE peptide functionalization improved penetration of HFn NPs in both G422 and GL261 glioma spheroids. Scale bar, 50 μm.


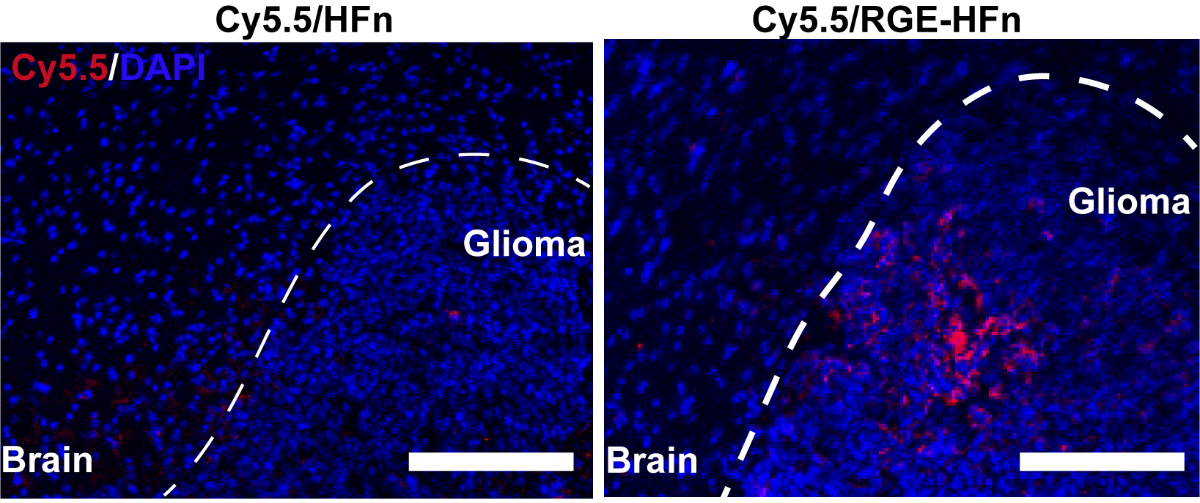


**Fig. S9.** Representative LSCM images of the excised brain tissue sections. Scale bar, 50 μm. (*n* = 5 biologically independent mice per group).


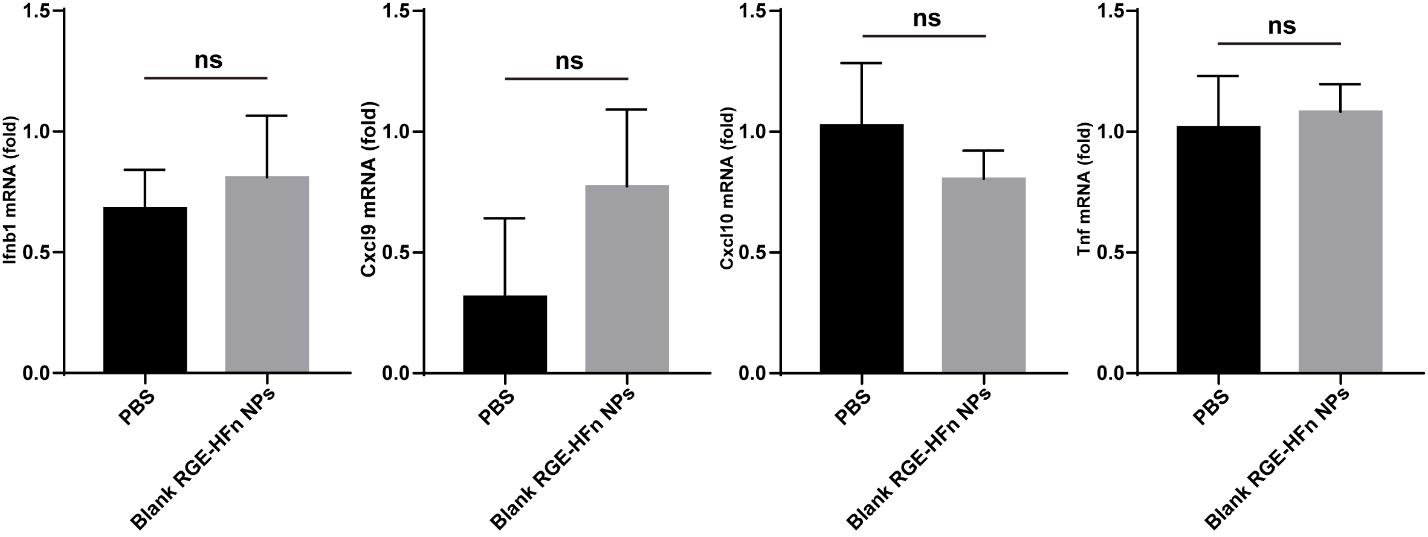


**Fig. S10.** qRT-PCR analysis of Ifnb1, Cxcl9, Cxcl10 and TNF-α mRNA expression in THP-1 cells after treatment with blank RGE-HFn NPs or PBS as controls (*n* = 3 biologically independent samples per group). *p* > 0.05 means no statistical significance (ns). Experiments were repeated twice independently with similar results. Significant differences were assessed using a two-tailed unpaired Student’s *t* test. Data are presented as mean ± SD from the second repeat.


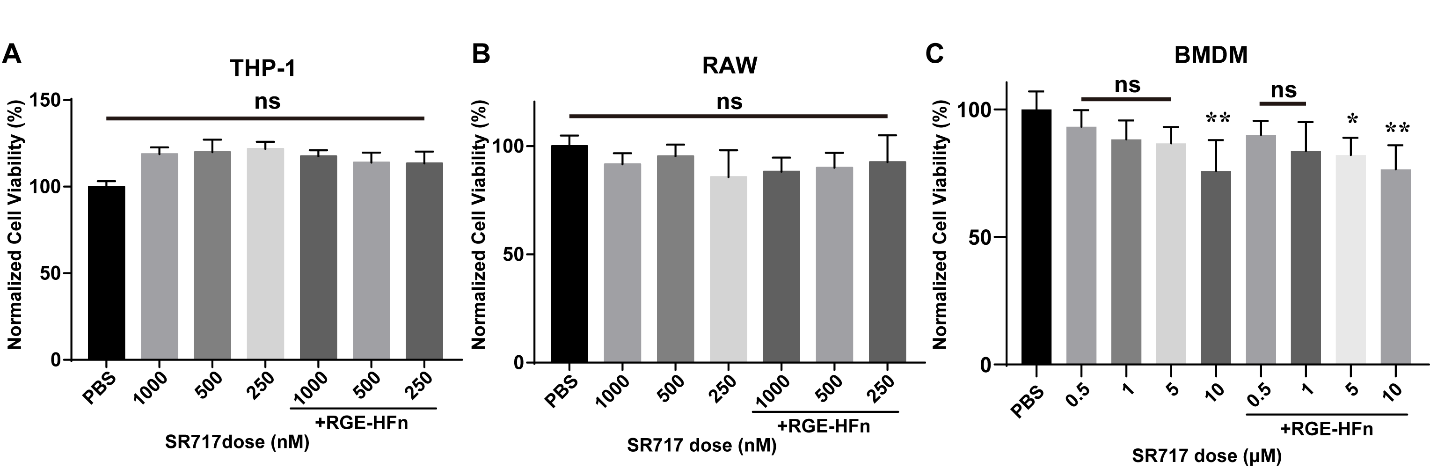


Fig. S11. (A-C) Cytotoxicity of SR717@RHE-HFn NPs in THP-1 (A), RAW (B) cell lines or bone marrow-derived monocytes (BMDMs) (C) (*n* = 6 biologically independent samples per group). *p* > 0.05 (ns), *p < 0.05 and **p < 0.01 compared to PBS. Experiments were repeated twice independently with similar results. Significant differences were assessed using a one-way ANOVA with Tukey test (A-C). Data in (A-C) are presented as mean ± SD from the second repeat.


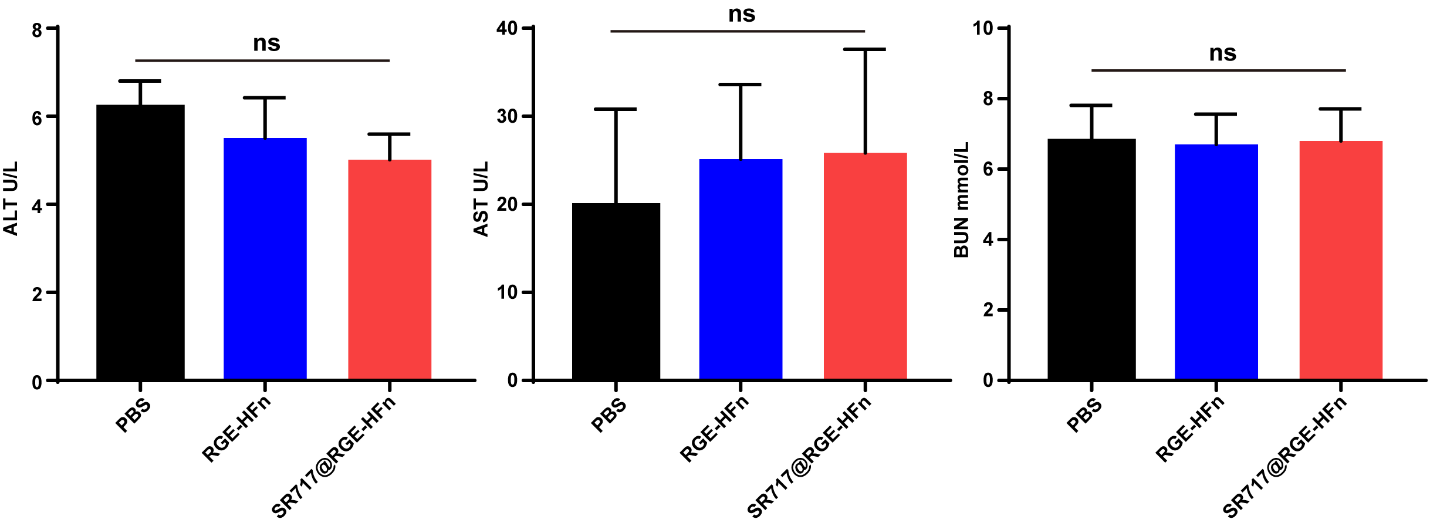


Fig. S12. Blood biochemical marker analysis of tumor-bearing mice treated with different regimens (*n* = 4 biologically independent mice per group). *p* > 0.05 (ns). Experiments were repeated twice independently with similar results. Significant differences were assessed using a one-way ANOVA with Tukey test. Data are presented as mean ± SD from the second repeat.


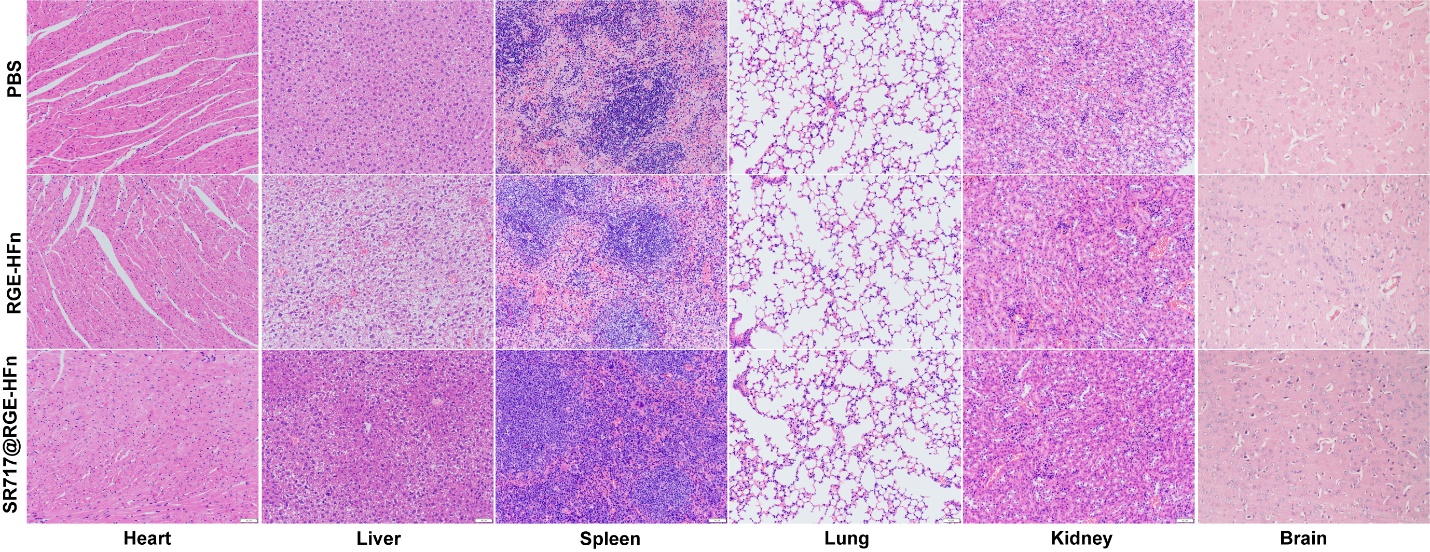


Fig. S13. H&E morphology evaluation of main organs in each treatment group. Scale bar, 100 μm.


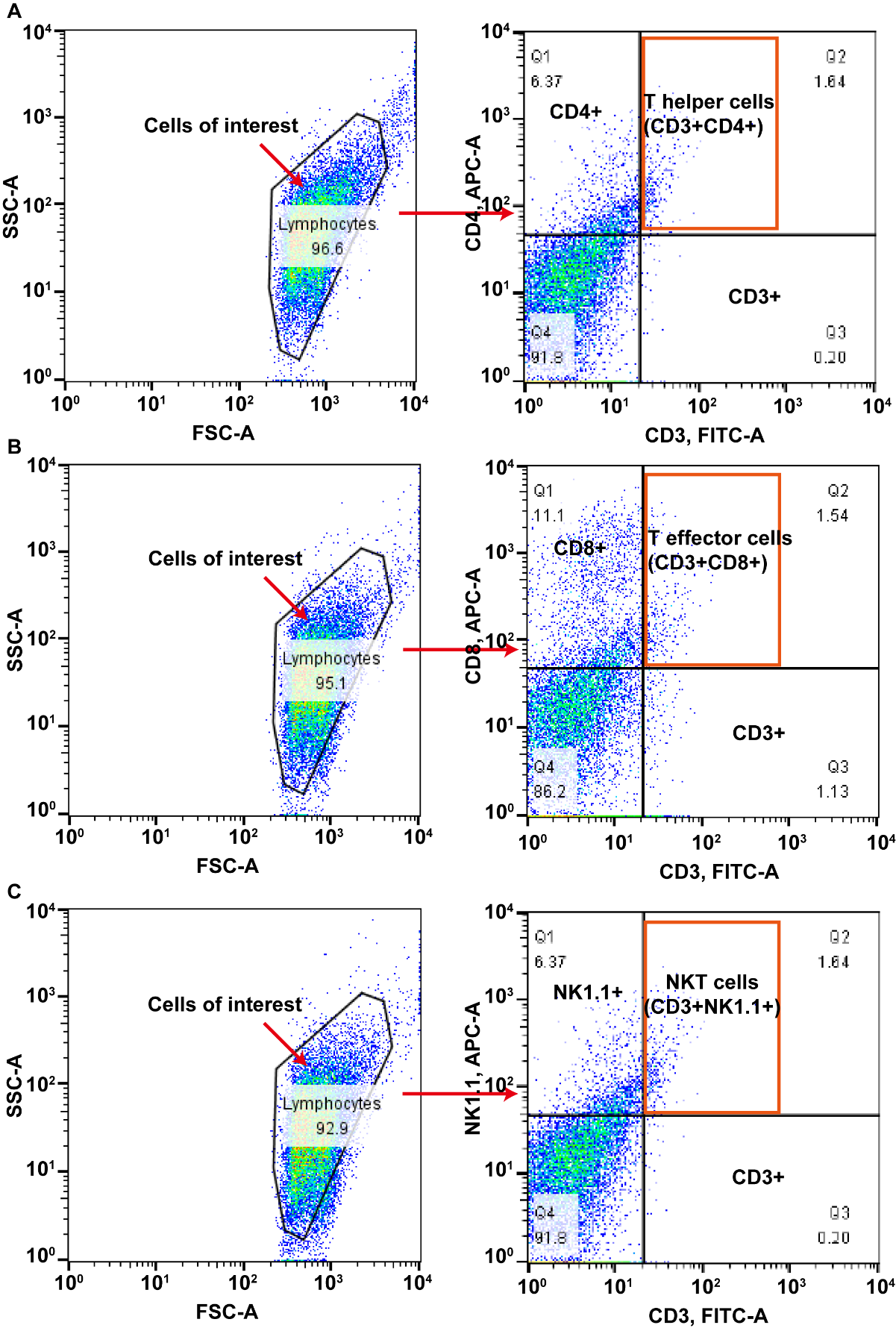


Fig. S14. Gating strategy used for flow cytometry analysis of T cells or NK cells. (A-C) Dissociated brain tumor cells were analyzed by flow cytometry to examine the tumor- associated T lymphocytes or NK cells. 50000 events were collected for each sample in the analysis. Cells of interest were chosen based on size in the FSC/SSC plots. Total T cell number was determined by CD3^+^ cell counts, and CD4^+^ T cells (A), CD8^+^ T cells (B) or NK1.1^+^ NK cells (C) were further gated within CD3^+^ T lymphocytes.

Table S1. Zeta potential characterization of HFn, Pep-1-HFn, CGKRK-HFn, RGE-HFn and SR717@RGE-HFn NPs.

| NPs | Zeta Potential (mV) |
| --- | --- |
| HFn NP | -7.3 |
| Pep-1-HFn NP | -9.4 |
| CGKRK-HFn NP | -7.9 |
| RGE-HFn NP | -6.9 |
| SR717@RGE-HFn NP | -6.8 |

Table S2. Primer sequences for qRT-PCR analysis.

| Gene name | Forward Primer sequence | Reverse Primer sequence |
| --- | --- | --- |
| Mouse Ifnb1 | GCCTTTGCCATCCAAGAGATGC | ACACTGTCTGCTGGTGGAGTTC |
| Mouse Cxcl9 | CCTAGTGATAAGGAATGCACGATG | CTAGGCAGGTTTGATCTCCGTTC |
| Mouse Cxcl10 | ATCATCCCTGCGAGCCTATCCT | GACCTTTTTTGGCTAAACGCTTTC |
| Mouse TNF-α | GGTGCCTATGTCTCAGCCTCTT | GCCATAGAACTGATGAGAGGGAG |
| Human Ifnb1 | CTTGGATTCCTACAAAGAAGCAGC | TCCTCCTTCTGGAACTGCTGCA |
| Human Cxcl9 | CTGTTCCTGCATCAGCACCAAC | TGAACTCCATTCTTCAGTGTAGCA |
| Human Cxcl10 | GGTGAGAAGAGATGTCTGAATCC | GTCCATCCTTGGAAGCACTGCA |
| Human TNF-α | CTCTTCTGCCTGCTGCACTTTG | ATGGGCTACAGGCTTGTCACTC |

Table S3. Antibody panel for spectral flow cytometry analyses

T cells flow panel

| Marker | CD3 | CD4 | CD8 |
| --- | --- | --- | --- |
| Antigen location | Extracellular | Extracellular | Intracellular |
| Fluorophore | FITC | APC | APC |
| Manufacturer & Cat. No. | Biolegend 100236 | Biolegend 100412 | Biolegend 126614 |
| Dilution | 1:100 | 1:100 | 1:100 |

NK cells flow panel

| Marker | CD3 | NK1.1 |
| --- | --- | --- |
| Antigen location | Extracellular | Extracellular |
| Fluorophore | FITC | APC |
| Manufacturer & Cat. No. | Biolegend 100236 | APC-65138 |
| Dilution | 1:100 | 1:100 |
